# Apathy and impulsivity in frontotemporal lobar degeneration syndromes

**DOI:** 10.1093/brain/awx101

**Published:** 2017-05-09

**Authors:** Claire J. Lansdall, Ian T. S. Coyle-Gilchrist, P. Simon Jones, Patricia Vázquez Rodríguez, Alicia Wilcox, Eileen Wehmann, Katrina M. Dick, Trevor W. Robbins, James B. Rowe

**Affiliations:** 1 Department of Clinical Neurosciences, University of Cambridge, UK; 2 University Medical Centre Hamburg-Eppendorf, University of Hamburg, Germany; 3 The Dementia Research Centre, Institute of Neurology, University College London, UK; 4 Behavioural and Clinical Neuroscience Institute, University of Cambridge, UK; 5 Department of Psychology, University of Cambridge, UK; 6 MRC Cognition and Brain Sciences Unit, 15 Chaucer Road, Cambridge, UK

**Keywords:** apathy, impulsivity, frontotemporal lobar degeneration, principal component analysis, voxel based morphometry

## Abstract

Apathy and impulsivity are common and disabling consequences of frontotemporal lobar degeneration. They cause substantial carer distress, but their aetiology remains elusive. There are critical limitations to previous studies in this area including (i) the assessment of either apathy or impulsivity alone, despite their frequent co-existence; (ii) the assessment of behavioural changes within single diagnostic groups; and (iii) the use of limited sets of tasks or questions that relate to just one aspect of these multifactorial constructs. We proposed an alternative, dimensional approach that spans behavioural and language variants of frontotemporal dementia, progressive supranuclear palsy and corticobasal syndrome. This accommodates the commonalities of apathy and impulsivity across disorders and reveals their cognitive and anatomical bases. The ability to measure the components of apathy and impulsivity and their associated neural correlates across diagnostic groups would provide better novel targets for pharmacological manipulations, and facilitate new treatment strategies and strengthen translational models. We therefore sought to determine the neurocognitive components of apathy and impulsivity in frontotemporal lobar degeneration syndromes. The frequency and characteristics of apathy and impulsivity were determined by neuropsychological and behavioural assessments in 149 patients and 50 controls from the PIck’s disease and Progressive supranuclear palsy Prevalence and INcidence study (PiPPIN). We derived dimensions of apathy and impulsivity using principal component analysis and employed these in volumetric analyses of grey and white matter in a subset of 70 patients (progressive supranuclear palsy, *n* = 22; corticobasal syndrome, *n* = 13; behavioural variant, *n* = 14; primary progressive aphasias, *n* = 21) and 27 control subjects. Apathy and impulsivity were present across diagnostic groups, despite being criteria for behavioural variant frontotemporal dementia alone. Measures of apathy and impulsivity frequently loaded onto the same components reflecting their overlapping relationship. However, measures from objective tasks, patient-rated questionnaires and carer-rated questionnaires loaded onto separate components and revealed distinct neurobiology. Corticospinal tracts correlated with patients’ self-ratings. In contrast, carer ratings correlated with atrophy in established networks for goal-directed behaviour, social cognition, motor control and vegetative functions, including frontostriatal circuits, orbital and temporal polar cortex, and the brainstem. Components reflecting response inhibition deficits correlated with focal frontal cortical atrophy. The dimensional approach to complex behavioural changes arising from frontotemporal lobar degeneration provides new insights into apathy and impulsivity, and the need for a joint therapeutic strategy against them. The separation of objective tests from subjective questionnaires, and patient from carer ratings, has important implications for clinical trial design.

## Teaser

Lansdall *et al*. reveal the structural brain changes leading to different types of apathy and impulsivity, across the spectrum of disorders caused by frontotemporal lobar degeneration, including frontotemporal dementia, progressive supranuclear palsy and corticobasal syndrome. The positive correlation between apathy and impulsivity indicates the need for a unified therapeutic strategy.

## Introduction

The clinical syndromes associated with frontotemporal lobar degeneration (FTLD) are clinically, genetically and pathologically heterogeneous (Josephs, 2008*a*; Piguet *et al.*, 2011; Rohrer and Warren, 2011). The syndromes include behavioural variant and language variants of frontotemporal dementia (FTD), progressive supranuclear palsy (PSP) and the corticobasal syndrome (CBS). Apathy and impulsivity are common and distressing features of these disorders (Zamboni *et al.*, 2008; Piguet *et al.*, 2011; Leroi *et al.*, 2012). They are diagnostic criteria for behavioural variant frontotemporal dementia (bvFTD) (Rascovsky *et al.*, 2011), and supportive criteria for PSP (Litvan *et al.*, 1996), but occur frequently across the full spectrum of disorders associated with FTLD (Mendez *et al.*, 2008; Burrell *et al.*, 2014; Coyle-gilchrist *et al.*, 2016). Apathy and impulsivity may be concurrent in an individual patient (Kertesz *et al.*, 2005; Chow *et al.*, 2009), suggesting that they are not simply opposite ends of a behavioural spectrum (Sinha *et al.*, 2013).

Both apathy and impulsivity are multifaceted constructs (Levy and Dubois, 2006; Dalley *et al.*, 2011; Nombela *et al.*, 2014), with multiple contributory factors. These factors may be expressed in terms of brain network pathology (Everitt and Robbins, 2005; Zamboni *et al.*, 2008; Eslinger *et al.*, 2013; Sinha *et al.*, 2013; Nombela *et al.*, 2014; Ye *et al.*, 2014, 2016), or the cognitive processes of motivation, reward and decision-making (Levy and Dubois, 2006; Adam *et al.*, 2012; Ahearn *et al.*, 2012; Zhang *et al.*, 2016), and pharmacology (Cools *et al.*, 2005; Eagle *et al.*, 2008; Dalley *et al.*, 2011). For example, apathy has been linked to deficits in motivational circuitry, specifically orbitofrontal connections to the ventral striatum (Everitt and Robbins, 2005; Levy and Dubois, 2006), and dopamine (Adam *et al.*, 2013; Sinha *et al.*, 2013). Impulsivity has also been linked to disruptions of dopaminergic, noradrenergic and serotonergic regulation of frontostriatal circuits (Dalley *et al.*, 2011; Ye *et al.*, 2014, 2015; Hughes *et al.*, 2015; Zhang *et al.*, 2016). The presence of apathy and impulsivity across different clinical diagnoses, and the evidence for their distinct components, creates a major challenge for the development of new therapeutic strategies.

To elucidate the physiological, pharmacological and genetic causes of apathy and impulsivity, and to design appropriately stratified and powered clinical trials of candidate treatments, three critical items are needed. First, a clear definition of the cognitive and behavioural components of apathy and impulsivity, from which to develop robust and targeted assessment tools. Second, knowledge of how these different components are represented transdiagnostically, across disorders associated with FTLD. Despite recent progress in clinical, pathological and genetic fractionation of these disorders (Gorno-Tempini *et al.*, 2011; Rascovsky *et al.*, 2011; Armstrong *et al.*, 2013), phenotypic boundaries are not always distinct. Third, one requires evidence for the neural basis of the components, both to generate surrogate markers in experimental medicines studies and to validate preclinical models of behavioural disorders.

Previous studies have often focused on apathy or impulsivity in isolation, employing a limited range of measures or summary metrics. However, apathy and impulsivity are co-existent multifactorial constructs, with each factor likely reflecting different anatomical and/or pharmacological underpinnings. A dimensional approach, such as a principal component analysis on a broad range of assessment types, would provide greater power to capture all aspects of apathy and impulsivity. We aimed to assess apathy and impulsivity from the patient and carer perspective, as measured by questionnaires of the type commonly used in clinical trials, enabling assessment of potential discrepancies between carer and patient ratings. We also used objective neuropsychological and behavioural tests to bridge between preclinical and clinical studies, supporting translational models. Taken together, these assessment tools capture the major domains of apathy and its principal confounds, including motivation, anhedonia, depression/mood and akinesia and the major domains of impulsivity, including reward sensitivity, response inhibition and information sampling.

It has also been common to study apathy or impulsivity in single diagnostic groups. However, the soft boundaries between clinical phenotypes and the overlap of clinical features as disease progresses (Kertesz *et al.*, 2005; Coyle-gilchrist *et al.*, 2016) calls for an alternative approach, accommodating commonalities across disorders. Such a transdiagnostic approach remains sensitive to the heterogeneity both within and across groups. For example, two patients with bvFTD can meet diagnostic criteria without sharing a single core clinical feature (Rascovsky *et al.*, 2011). In contrast, patients with the semantic variant of progressive aphasia (svPPA) meet different diagnostic criteria to bvFTD, but they often develop similar behavioural changes later in their disease course. Examination of the commonalities across the full spectrum of clinical phenotypes associated with FTLD therefore provides increased power and facilitates the dissection of major components of apathy and impulsivity.

This study drew on a dimensional approach. It was inspired by the ‘Research Domain Criteria’ framework for psychiatric disorders (Kozak and Cuthbert, 2016), which aims to develop new ways of classifying disorders based on dimensions of observable behaviour and neurobiological measures; embracing the overlap between clinical features in contrast to a categorical approach to diagnosis (Cuthbert, 2014). It can provide a mechanistic model to bridge between different levels of analysis of disease pathogenesis and their causal relationships. We implemented a data-driven analysis to identify the components (as dimensions) of apathy and impulsivity empirically, which we then interpret in terms of motivation, reward sensitivity, motor and cognitive control. Our specific hypotheses were that (i) apathy and impulsivity are multifactorial constructs, but with common and overlapping features; (ii) subjective and objective measures relate to the same components; and (iii) distinct frontostriatal, frontotemporal and brainstem circuits support the components of apathy and impulsivity.

## Materials and methods

### Context and participants

The Pick’s disease and Progressive supranuclear palsy Prevalence and INcidence (PiPPIN) study, provided the ideal arena to test our hypotheses, combining neuropsychological, behavioural and MRI assessments. This epidemiological study of FTD, CBS and PSP patients was carried out in the UK (Coyle-gilchrist *et al.*, 2016). Diagnoses were based on current criteria for bvFTD (Rascovsky *et al.*, 2011), primary progressive aphasia (PPA) syndromes (Gorno-Tempini *et al.*, 2011), PSP (Bensimon *et al.*, 2009) and CBS (Armstrong *et al.*, 2013), following clinical interview, physical examination, relevant exclusionary tests and brain imaging. The PPAs were subtyped (Gorno-Tempini *et al.*, 2011) into the non-fluent agrammatic variant (nvPPA), the semantic variant (svPPA), and a third group that included logopenic variant (lvPPA) and mixed aphasia (PPA as the prominent syndrome but not fitting criteria for one of the three defined subtypes). We estimated the years from symptom onset, based on recall of initial relevant symptoms. Two hundred and four patients were identified, 167 of whom were assessed in person by a member of the study team. Eighteen either died before neuropsychological assessment or were unable to undertake testing over and above diagnostic confirmation, leaving 149 patient datasets for analysis by principal component analysis. Fifty healthy age- and sex-matched controls were recruited from the Medical Research Council’s Cognition and Brain Sciences Unit volunteer panel, with no significant neurological or psychiatric history.

A subset of 70 patients (PSP, *n* = 22; CBS, *n* = 13; bvFTD, *n* = 14; nvPPA, *n* = 12; svPPA, *n* = 4; other PPA, *n* = 5) and 27 control subjects underwent MRI. The imaging subset was representative of the cohort, with no significant differences between the imaging subset (*n* = 70) and the non-imaged patients (*n* = 79) in terms of demographics, disease characteristics and the major outcome variables included in the analysis (Supplementary Table 1). Most patients underwent MRI on the same day as cognitive assessment (median and mode = 0 days).

Participants were tested while on their usual medication. Forty per cent of patients were taking antidepressant medications (for either affective or behavioural indications), 4% were taking antipsychotic medication, and 29% were taking dopaminergic medication (for movement disorder). Thirty-seven per cent were taking other medications that may act on the CNS including benzodiazepines (for anxiolysis, sedation or myoclonus), antiepileptic medication, analgesics (opioid, gabapentin, pregabalin), including one case on cholinesterase inhibitors.

The study was approved by the Cambridge 2 Research Ethics Committee. Informed consent was obtained at each study visit, with a ‘personal consultee’ process used for participants who lacked mental capacity, in accordance with UK law.

### Neuropsychological, behavioural and imaging assessment battery

In selecting our test battery, we applied the following principles: to include clinically standard tests as well as experimental paradigms; to include questionnaires to be completed by patients as well as by carers so as to provide complementary perspectives; to include both subjective symptom-based questionnaires and objective neuropsychological tests for both patients and controls; to measure depression symptoms and akinesia as well as direct tests of cognitive and behavioural aspects of apathy and impulsivity; to prioritize untimed tests in view of likely akinesia in many participants; and use only tasks that have been published and used with independent cohorts. Details of the questionnaires and behavioural tasks used to evaluate apathy and impulsivity are listed in Table 1, while we summarize below the less common assessment tools (see Supplementary material for full details).

**Table 1:** Assessment battery

Measurement	Type	Rater	Description	Outcome variables entered for local PCA	Summary of scores or local PCA loadings
Apathy Evaluation Scale (AES)	Q	P, I, C	18 items assessing emotional, behavioural and cognitive constructs of apathy	Cognition Emotion Behaviour	AES 1: patient ratings AES 2: mainly carer and clinician
Barratt Impulsiveness Scale (BIS)	Q	P	30 item self-report questionnaire. Reflecting the multifactorial structure of impulsivity.	Attention Motor Self control Cognitive complexity Perseverance Cognitive instability	BIS 1: Attention, self control, cognitive com plexity, perseverance. BIS 2: Motor and cognitive instability
Behavioural Inhibitory System Behavioural Approach System (BIS/BAS)	Q	P	24 item self-report questionnaire based on Grey’s biopsychological theory of personality	BIS subscore BAS drive BAS fun-seeking BAS reward Responsiveness	BIS/BAS 1: BAS subscores BIS/BAS 2: BIS subscore
Cambridge Behavioural Inventory (CBI-R)	Q	C	45 item questionnaire, developed to evaluate behavioural changes associated with dementia.	Memory/orientation Everyday skills; self-care Abnormal behaviour; mood; Beliefs; Eating habits; sleep; Stereotypical behaviour; Motivation	CBI 1: Challenging behaviours CBI 2: Everyday skills and self-care
Motivation and Energy Inventory (MEI)	Q	P	27 item questionnaire developed to evaluate reductions in motivation and energy in depression research, although commonly used in other disease areas.	Total score	Total score
Snaith-Hamilton Pleasure Scale (SHAPS)	Q	P	14 item questionnaire targeting hedonic capacity (anhedonia).	Total score	Total score
Beck Depression Inventory (BDI)	Q	P	21 item questionnaire, widely used to measure the severity of depression.	Total score	Total score
Kirby	Q	P	Serial forced choice questionnaire to quantify the tendency to prefer small immediate rewards over larger delayed rewards	K_diff_ calculated: Difference in delayed discounting (K) from small to large delayed rewards (K_large_-K_small_), termed K_diff_.	K_diff_ single score
Information Sampling Task (IST)	B	P	Reflection impulsivity task, based on the information and time used by participants before making a two-choice probabilistic decision.	Proportion of correct trials Box latency; colour latency; Total correct; sampling error	IST 1: Proportion of correct trials, boxes opened, total correct IST 2: Box and colour latency IST 3: Sampling error, - boxes opened
Cued reinforcement reaction time (CRRT)	B	P	Reward sensitivity task measuring motivationally driven behaviour.	Speeding first half of trials Speeding second half of trials Difference in speeding from FH-SH Total errors	CRRT 1: Difference speeding, Errors, - Speeding FH CRRT 2: Speeding SH, Difference speeding
Stop signal task (SST)	B	P	Action cancellation task.	SSRT Median reaction time on correct GO trials Proportion of successful stops	SST 1: all variables entered
Motor NoGo	B	P	Inhibition of a prepotent motor response.	Calculated Dprime: lower values reflect decreased ‘hits’ (correct on Go trials) and increased false alarms (Go on NoGo trials: commission errors).	Dprime
Saccade NoGo	S	P	Inhibition of cued saccade	Calculated Dprime: lower values reflect decreased ‘hits’ (correct on Go trials) and increased false alarms (Go on NoGo trials: commission errors).	Dprime
Cambridge Gambling Task (CGT)	B	P	Visual gambling task to measure risk-taking and decision making behaviour.	N/A	N/A

Test type included questionnaires (Q), behavioural tasks (B) and saccades (S). Tests were completed by the patient (P), carer (C) or investigator (I).

The assessment of cognitive impairment and disease severity included the Addenbrooke’s Cognitive Examination-Revised (ACE-R), Mini-Mental State Examination (MMSE), Frontotemporal Dementia Rating Scale (FRS), PSP Rating scale (PSPRS) and Frontal Assessment Battery (FAB).

The Cambridge Neuropsychological Test Automated Battery (CANTAB, Cambridge Cognition, UK) was used for the Stop-signal Task (Aron *et al.*, 2003), Information Sampling Task (Lawrence *et al.*, 2009) and the modified version of the Cambridge Gambling Task for clinical cohorts (Supplementary material). However, the gambling task was removed after 37 participants due to floor effects and difficult task engagement by patients. Patients were able to perform the Cued Reinforcement Reaction Time task, which provides an alternative measure of reward responsiveness (Cools *et al.*, 2005). Prior to each trial, participants observed a coloured rectangle signalling the probability of reward following a correct response (20% versus 80% probability). Participants then identified the ‘odd-one-out’ of three circles to receive feedback: 100 points for a fast correct, 1 point for a slow correct response and 0 points for an incorrect response. This task induces a ‘reinforcement-related speeding’ effect, making faster responses with increased probability of reward. Forty practice trials without feedback were used to titrate reaction time thresholds to individual differences in cognitive speed.

The saccadic NoGo task (Zhang *et al.*, 2016) used direct infrared oculography from a head-mounted saccodometer (OberConsulting). Each session included 300 trials, following 10 calibration trials. Participants fixated centrally (red/green dots) on a screen at ∼1.5 m distance. After 300 ms, one of the central cues was removed and a red dot was presented at −10 or +10 degree horizontal displacement (randomized, 50:50). In 50% of trials, the green central cue remained and participants responded by a saccade to lateral target (Go trials). In NoGo trials, the red central cue remained and participants refrained from making a saccade. Data were analysed using LatencyMeter (Ober Consulting Version 6.5), with automatic trials validation to eliminate abnormal saccades based on the position and velocity profile of each trace. The motor NoGo task was analogous to the saccadic task but used a joystick operated by the right hand (Supplementary material). Outcome measures for NoGo tasks included d-prime for performance accuracy, commission and omission error rates and reaction times.

MRI was performed at the Wolfson Brain Imaging Centre, using a TIM-Trio 3T scanner (Siemens). T_1_-weighted magnetization-prepared rapid acquisition gradient-echo (MPRAGE) images were acquired with a repetition time = 2300 ms, echo time = 2.86 ms, matrix = 192 × 192, in-plane resolution of 1.25 × 1.25 mm, 144 slices of 1.25 mm thickness, inversion time = 900 ms and flip angle = 9°. Significant effects were identified using cluster-level statistics. An uncorrected height threshold of *P* < 0.005 was used to identify voxels and spatial extent was corrected for multiple comparisons at *P* < 0.05. Preprocessing used diffeomorphic anatomical registration using exponentiated Lie algebra (DARTEL) in SPM12 following brain extraction. The T_1_ images were segmented using default settings to output the DARTEL import images for grey and white matter. Then a study-specific template was created using five age-matched participants from each of the seven diagnostic groups (to reduce group bias). The remaining subjects’ data were warped to the template. Next, the grey and white matter template segments were affine-transformed to MNI space. The affine template transformation was applied to the maps of the individual participants together with smoothing by an 8 mm isotropic full-width at half-maximum Gaussian kernel. The total intracranial volume was calculated using Tissue Volumes function in SPM12, and study-specific masks created from voxels with a value of >0.1 in >80% (Barnes *et al.*, 2010) of the images.

### Statistical analysis

The analyses aimed to (i) identify the underlying components of apathy and impulsivity; and (ii) determine their associated neural correlates. Statistical analysis of behavioural and neuropsychological data used SPSS v22.0 (IBM). Demographic data and disease characteristics, including age, gender, ACE-R total score and FRS total score, and the principal outcome measure for each of the eight questionnaires and four objective behavioural tasks were also compared using two-sample *t*-tests between groups (patient versus control).

#### Principal components analysis

Principal components analysis (PCA) identified the components of apathy and impulsivity that best explained the data variance, reducing the dimensionality and increasing reliability by combining data from multiple tests. PCAs were run on patient and control data combined (*n* = 199, noting that there were no major differences to the component structure if using only 149 patients’ data). The correlation matrix was used for extraction of components. Kaiser-Meyer-Olkin and Bartlett’s test of sphericity were used to determine the adequacy of the sample size for PCA analysis.

We took a hierarchical approach to the PCA, since many of the individual tasks give rise to multiple outcome measures (Supplementary Fig. 1). First, task-specific ‘local’ PCAs using orthogonal varimax rotation were performed separately on the individual questionnaires and behavioural measures (Table 1). Varimax rotation ensures orthogonality, maximizes the dispersion of loadings within components and facilitates interpretation. Selection of components used Kaiser’s or Cattell’s criteria, whichever was more inclusive, plus an additional criterion of explaining >10% of the variance. Component loadings >0.50 were considered meaningful and component scores were computed using the regression method. Second, the components extracted from each of the local PCAs were included in a final PCA, which also included total scores or d-prime from the tests that were not subject to local PCA (Table 1). Selection of components used Kaiser’s or Cattell’s criteria, whichever was more inclusive. Component scores were also correlated with age, cognition (ACE-R, MMSE and FAB) and disease severity (FRS) in SPSS v22 (IBM).

#### Voxel-based morphometry

Due to orthogonality of PCA components, their neural correlates were identified by a general linear model, using the smoothed normalized grey and white matter segments. The design matrix included the eight mean centred Principal Component Factor scores, age, gender and total intracranial volume and an intercept. Both positive and negative contrasts were examined from the General Linear Model for all eight principal components. Significant effects were identified using cluster-level statistics (FWEc *P* < 0.05, corrected for multiple comparisons) above a height threshold of *P* < 0.005 (uncorrected). The non-stationary cluster extent correction was applied in view of the non-uniformity of the data.

## Results

### Behavioural results

The neuropsychological and behavioural performance by patient and control data are presented in Tables 2 and 3 (see Supplementary Table 2 for performance by diagnostic group). Patients and controls were matched for age and gender. Patients had significant cognitive deficits compared to controls in addition to significantly higher apathy (Apathy Evaluation Scale, AES), impulsivity (Barratt Impulsiveness Scale, BIS), depression (Beck Depression Inventory, BDI) and anhedonia (Snaith-Hamilton Pleasure Scale, SHAPS) with lower levels of motivation (Motivation and Energy Inventory, MEI). Patients also demonstrated significant impairments on behavioural tasks of reflection impulsivity (information sampling task), incentive motivation (cued reinforcement), response inhibition (limb-motor and saccade tasks) and action cancellation (Stop-Signal task). The Behavioural Inhibition System/Behavioural Activation System (BIS/BAS) and Kirby responses did not differentiate between patients and controls.

**Table 2:** Summary of patient and control characteristics

Variable	Controls	Patients (all groups)	T statistic	Group difference
**Demographics and cognition**				
Age	70.6 ± 6.5	69.9 ± 8.2	0.9	NS
Gender M:F	23:27	76:73	(χ^2 ^= −0.6)	NS
ACE-R total (max. 100)	95.6 ± 4.4	64.7 ± 22.6	12.7	^**^(^*^)
MMSE total (max. 30)	29.3 ± 1.2	22.3 ± 6.8	9.6	^**^(^*^)
FRS % score (max. 100)	92.1 ± 10.8	37.9 ± 26.5	18.5	^**^(^*^)
**Questionnaires**				
Apathy Evaluation Scale (AES, max. 72):				
Carer	24.2 ± 5.7	48.1 ± 12.4	−16.7	^**^(^*^)
Patient	25.7 ± 5.6	36.1 ± 9.4	−7.8	^**^(^*^)
Clinician	25.9 ± 7.3	43.6 ± 10.0	−11.8	^**^(^*^)
Barratt Impulsiveness Scale (BIS, max. 120)	57.0 ± 7.4	63.6 ± 8.1	−4.6	^**^(^*^)
Behavioural Inhibition System/Behavioural Activation System (BIS/BAS):				
BIS subscore	19.9 ± 3.4	20.6 ± 4.5	−1.0	NS
BAS drive	10.0 ± 2.1	10.9 ± 3.2	−1.9	NS
BAS fun-seeking	10.7 ± 2.2	11.3 ± 3.0	−1.2	NS
BAS reward responsivness	15.8 ± 2.4	16.6 ± 2.7	−1.7	NS
Motivation and energy inventory (MEI, max. 144)	108.9 ± 17.2	81.1 ± 26.4	7.0	^**^(^*^)
Beck Depression Inventory (BDI, max. 63)	4.2 ± 4.0	13.0 ± 10.1	−6.7	^**^(^*^)
Snaith-Hamilton pleasure scale (SHAPS, max. 56)	18.6 ± 4.4	22.5 ± 4.8	−4.5	^**^(^*^)
Neuropsychiatric Inventory (NPI, fraction with positive response):				
Apathy subscore	0.000 ± 0.0	0.616 ± 0.5	−13.3	^**^(^*^)
Disinhibition subscore	0.020 ± 0.1	0.336 ± 0.5	−6.5	^**^(^*^)
Cambridge Behavioural Inventory (CBI-R, max 180)	5.2 ± 5.6	66.7 ± 35.2	−18.2	^**^(^*^)
Kirby (difference)	0.005 ± 0.04	0.019 ± 0.1	−1.6	NS
**Behavioural tasks**				
Information Sampling Task (IST)				
Probability of being correct Fixed	0.866 ± 0.1	0.747 ± 0.1	4.9	^**^(^*^)
Probability of being correct Decreasing	0.806 ± 0.1	0.668 ± 0.2	5.4	^**^(^*^)
Cued reinforcement reaction time (CRRT)				
Reward-related speeding	−43.4 ± 90.9	196.3 ± 739.1	−2.4	^*^
Total errors	3.8 ± 3.4	4.2 ± 5.7	−0.5	NS
Cambridge Gambling Task				
Deliberation time	2240.0 ± 767	7053.0 ± 4449	1.4	^**^(^*^)
Risk adjustment	1.57 ± 1.1	0.23 ± 0.9	4.1	^**^(^*^)
Stop Signal Task (SST)				
Stop signal reaction time (SSRT)	181.1 ± 41.7	439.8 ± 190.4	−3.1	^**^(^*^)
Motor Go/NoGo Dprime	4.4 ± 0.3	3.2 ± 1.3	7.8	^**^(^*^)
Saccade Dprime	2.4 ± 0.9	0.75 ± 1.1	7.4	^**^(^*^)

Objective measures corrected for outliers ± 3 standard deviations (SD) of the mean. Independent samples *t*-test uncorrected for multiple comparisons are shown outside parentheses: ^**^P < 0.001, ^*^P < 0.05. NS = not significant. Significance after Bonferroni correction is indicated by (^*^). Note that some measures are not independent, e.g. MMSE is a component of the ACE-R, and NPI subscales are component of the total NPI score. CGT task data from 37 participants only.

**Table 3:** Demographics, cognitive, functional and motor features by diagnosis

	PSP	CBS	svPPA	PPA	bvFTD	nvPPA	Control
*n*	41	37	12	11	32	16	50
Age	72.9 ± 8.5	69.7 ± 7.8	71.1 ± 4.1	73.1 ± 4.9	64.0 ± 7.3	71.6 ± 9.1	70.6 ± 6.5
Gender (M:F)	21:20	18:19	7:5	5:6	18:14	7:9	23:27
Duration (of symptoms)	4.5 ± 3.4	4.1 ± 2.3	5.7 ± 2.9	4.1 ± 2.2	4.9 ± 3.0	2.0 ± 2.0	NA
ACE-R (max. 100)	75.5 ± 14.6	65.7 ± 21.3	29.2 ± 14.7	58.5 ± 20.5	59.0 ± 26.9	64.4 ± 21.0	95.6 ± 4.4
MMSE (max. 30)	25.0 ± 4.8	22.0 ± 6.6	11.8 ± 8.7	21.0 ± 5.1	21.4 ± 7.6	23.0 ± 6.3	29.3 ± 1.2
FRS % score (max. 100)	40.9 ± 25.1	31.4 ± 23.3	20.9 ± 14.6	66.3 ± 28.4	26.8 ± 18.0	63.7 ± 28.4	92.1 ± 10.8
FAB (max. 18)	10.5 ± 4.0	10.0 ± 4.4	9.4 ± 3.8	10.0 ± 4.4	9.4 ± 5.3	9.2 ± 4.4	16.8 ± 1.2
PSP-RS (max. 100)	43.8 ± 14.8	39.6 ± 16.1	NA	5.3 ± 4.7	16.1 ± 10.0	8.4 ± 6.2	NA
Akinesia, *n* (%)	35	27	2	2	22	31	0
Rigidity, *n*	35	27	0	1	6	1	0
Dystonia, *n*	25	24	0	0	2	0	0
Apraxia, *n*	22	33	2	8	8	11	0
Vertical gaze palsy^a^, *n*	41	19	0	2	3	1	0
Postural instability/falls^b^, *n*	41	24	0	1	7	2	0
Myoclonus, *n*	3	22	0	3	3	5	0

^a^Or slowing of vertical saccades; ^b^or wheelchair dependence.

MMSE = Mini-Mental State Examination; FAB = Frontal Assessment Battery (FAB); PSP-RS = progressive supranuclear palsy rating scale.

Patients also demonstrated cognitive and functional impairment across groups compared to controls, as measured by the ACE-R, MMSE, FRS, and FAB (Table 3). Additional motor features were also present in some patients across diagnostic groups, including akinesia, rigidity, dystonia, apraxia, vertical gaze palsy, postural instability, and myoclonus (Table 3).

### Principal component analysis

The sample size was adequate for analysis (Kaiser-Meyer-Olkin stat = 0.743) and correlations between items were sufficiently large for PCA (Bartlett’s test of sphericity_231_ = 508.013, *P* < 0.001). Eight components were extracted from the final PCA (eigenvalues range: 1.039–4.963). The rotated component matrix is provided in Table 4. Note that assessments that are traditionally considered to be associated with apathy and impulsivity load onto the same factors (e.g. AES and BIS), reflecting a high positive correlation between components of apathy and components of impulsivity. Inclusion of the Cambridge Gambling task data from 37 participants did not alter the factor structure, but in view of limited numbers this test was removed from the main analyses.

**Table 4:** Rotated component matrix extracted from principal components analysis

Input variable	Component structure							
	Component 1 Patient-rated change	Component 2 Carer-rated change: Everyday skills and self-care	Component 3 Carer-rated change: Challenging behaviours	Component 4 Impulsive behaviours	Component 5 Impulsivity self-report	Component 6 Goal-directed decision- making	Component 7 Stop Signal Task	Component 8 Outcome sensitivity
**Eigenvalue**	**4.963**	**2.183**	**1.664**	**1.514**	**1.385**	**1.186**	**1.111**	***1.039***
AES 1	**0.832**	−0.069	−0.121	0.151	−0.078	−0.003	−0.041	−0.069
BIS 1	**0.735**	0.086	0.083	0.221	0.080	−0.003	−0.095	−0.052
BDI-T	**0.756**	0.345	0.100	0.073	0.158	0.097	−0.026	−0.030
MEI-T	**−0.837**	−0.232	−0.061	−0.109	−0.023	0.034	0.142	0.007
SHAPS-T	**0.688**	0.147	0.281	−0.067	−0.276	−0.136	0.068	0.075
AES 2	0.067	**0.714**	**0.529**	0.074	0.035	0.006	−0.110	−0.151
CBI 2	0.233	**0.831**	−0.084	0.151	−0.113	0.023	−0.155	0.042
NPI-A	0.192	**0.705**	0.355	0.119	−0.086	0.048	0.029	−0.050
CBI 1	0.035	0.118	**0.880**	0.078	0.104	−0.135	−0.066	−0.069
2NPI-D	0.135	0.083	**0.825**	−0.008	−0.017	0.039	0.017	0.092
IST 2	0.170	0.030	−0.037	**0.683**	−0.128	0.365	−0.166	0.006
CRRT 1	0.007	0.014	−0.006	**0.658**	−0.013	−0.104	0.390	0.109
Go/NoGo	−0.259	−0.135	−0.113	**−0.642**	0.130	0.042	0.259	0.007
Saccades	−0.162	−0.198	−0.081	**−0.530**	−0.319	0.221	0.018	0.158
BIS 2	0.022	−0.121	−0.015	−0.100	**0.841**	−0.023	−0.065	0.077
BISBAS 1	−0.198	−0.005	0.265	0.083	**0.631**	0.375	−0.209	−0.011
IST 1	−0.188	−0.204	−0.080	−0.177	0.013	**0.556**	0.311	0.052
CRRT 2	0.084	0.162	−0.037	0.063	0.078	**0.725**	−0.031	−0.078
SST 1	0.183	0.109	0.021	0.044	0.167	−0.087	**−0.793**	0.030
BISBAS 2	0.068	0.090	−0.088	0.042	0.242	−0.179	0.141	**0.804**
Kirby	0.199	0.230	−0.126	0.040	0.220	−0.151	0.215	**−0.658**
IST 3	0.255	0.382	−0.198	−0.167	0.335	−0.007	0.283	−0.001

Numbers (1, 2, 3) indicate the different components extracted from local PCA for AES, CBI, BIS, BIS/BAS, IST, SST, CRRT. Additional input variables included the total score for BDI, MEI and SHAPS, NPI apathy and disinhibition subscores, Kirby difference value representing the difference in delayed discounting for low versus high rewards and Dprime performance accuracy values for Go/NoGo tasks. High scores on Components 1–5 and 8 indicate worse performance, while low scores on Components 6 and 7 indicate worse performance. Factor loadings >0.5 are highlighted in bold.

Short names for components were given that encapsulate their strongly weighted processes or tasks. However, please refer to Table 4 for the weighting of each questionnaire or test to each component. Component 1 reflects patient ratings on questionnaires of apathy, impulsivity and related changes termed ‘Patient-rated change’. Higher scores indicate increased questionnaire endorsement of apathy, impulsivity, depression, anhedonia and low motivation. Components 2 and 3 are associated with carer ratings of patient change, with higher scores reflecting more abnormal behaviours. Component 2, termed ‘Carer-rated change in everyday skills and self-care’, is weighted to carer AES, Cambridge Behavioural Inventory (CBI) (everyday skills, self-care, sleep and motivation) and the Neuropsychiatric Inventory (NPI) apathy subscore. The carer AES also loads onto Component 3 ‘Carer-rated change in complex behaviours’, in addition to remaining subscores of the CBI (abnormal behaviour, eating habits, stereotypic behaviours) and the NPI-disinhibition. The final questionnaire-based component, Component 5, is termed ‘Impulsivity self-report’, to reflect increased ratings on BIS motor and cognitive instability and BIS/BAS subscores.

Component 4 is associated with poor performance on NoGo, information sampling and cued reinforcement tasks, and termed ‘Impulsive behaviours’. Higher scores on Component 6, termed ‘Goal-directed decision-making’, represent accurate performance on the information sampling task and sensitivity to reward on the cued reinforcement task. On Component 7, termed ‘SST performance’, high scores reflect shorter stop-signal reaction times. Component 8, termed ‘Outcome sensitivity’, captures the incentive motivation elements of the Kirby and behavioural avoidance of the BIS/BAS. Higher scores reflect reduced difference in temporal discounting from small to large values of *K* on the Kirby and increased behavioural avoidance.

**Figure 1:**
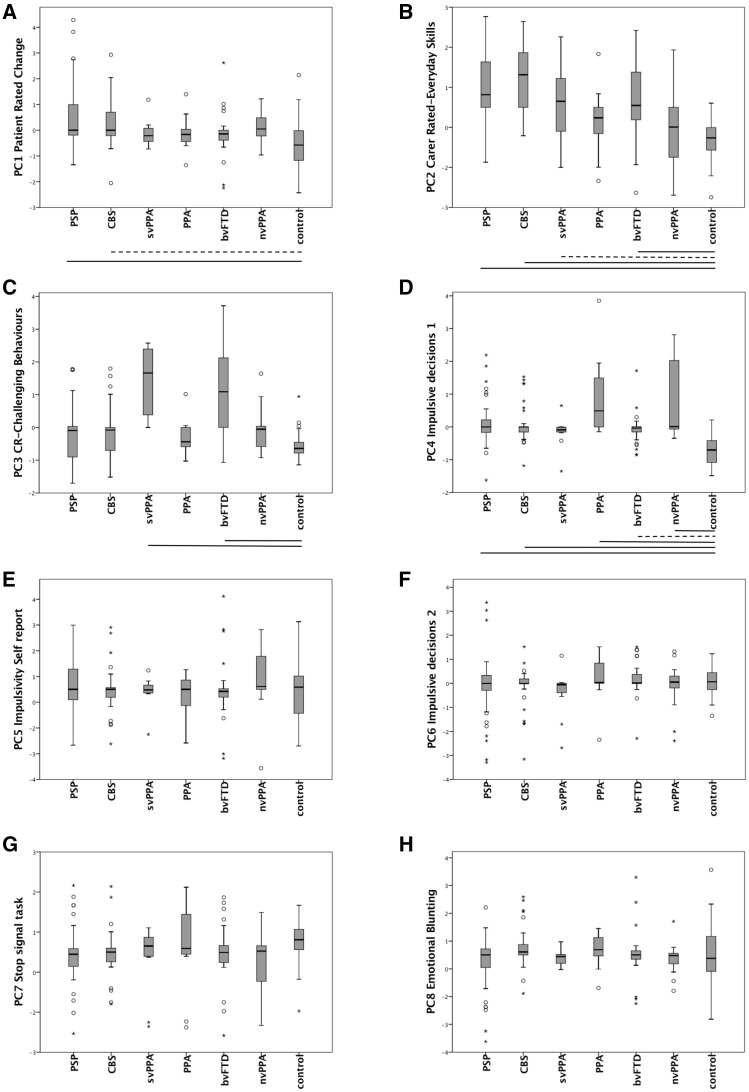
**Box plots of component scores (1–8) by diagnosis.** Scale bars indicate *post hoc* Tukey tests for each group versus controls (thick: *P* < 0.001, dotted: *P* < 0.05). Significant changes were observed for (**A**) PSP, CBS versus controls, (**B**) PSP, CBS, svPPA, bvFTD versus controls, (**C**) svPPA, bvFTD versus controls, and (**D**) PSP, CBS, PPA, bvFTD, nvPPA versus controls. Box plots **E–H** showed no significant differences. PC = principal component. ^*^Extreme outlier (3 × interquartile range, IQR), o = mild outlier (1.5 × IQR).

The components were not specific to individual disease groups, but reflected the transdiagnostic nature of apathy and impulsivity. Figure 1 shows the distribution of component scores in each of the six patient groups and controls. ANOVAs confirmed a significant effect of group (and *post hoc t*-tests comparing each patient group to controls) with respect to Component 1 [*F*(6 192) = 6.35, *P* < 0.001: *post hoc* control versus PSP *P* < 0.001, versus CBS *P* < 0.05]; Component 2 [*F*(6 192) = 17.1, *P* < 0.001: *post hoc* control versus PSP *P* < 0.001, versus CBS *P* < 0.001, versus bvFTD *P* < 0.001, versus svPPA *P* < 0.05]; Component 3 [*F*(6 192) = 19.9, *P* < 0.001: *post hoc* control versus bvFTD *P < *0.001, versus svPPA *P* < 0.001]; Component 4 [*F*(6 192) = 15.9, *P* < 0.001: *post hoc* control versus PSP *P* < 0.001, versus CBS *P* < 0.001, versus PPA *P* < 0.001, versus bvFTD *P* < 0.05, versus nvPPA *P* < 0.001]; Component 5 [*F*(6 192) < 1], Component 6 [*F*(6 192) < 1], Component 7 [*F*(6 192) = 1.7, ns]; and Component 8 [*F*(6 192) = 2.0, *P* = 0.07].

Parametric Pearson’s correlation analyses (Table 5) revealed that the patient rated change component (Component 1) was related to disease severity (FRS) and frontal dysfunction (FAB). Higher scores on Components 2–4 correlated with more severe disease (FRS), greater cognitive decline (ACE-R, MMSE) and frontal dysfunction (FAB). Component 2 was positively correlated with the PSP-RS, reflecting greater PSP-like cognitive and motor impairment. Poor performance on behavioural impulsivity tasks (Component 4) was negatively correlated with PSP-RS. Executive function, measured by ACE-R fluency, correlated with Components 1–4 and 7.

**Table 5:** Pearson’s correlations between the eight orthogonal components identified by principal components analysis and the patients’ demographic, cognitive and severity ratings

Component	Age	FRS %	ACE-R	ACE-R fluency	MMSE	PSP-RS	FAB
(PC1) Patient-rated change	0.050	−0.271^**^	−0.125	−0.277^**^	−0.085	0.134	−0.258^*^
(PC2) Carer-rated change: everyday skills and self-care	−0.047	−0.658^**^	−0.343^**^	−0.335^**^	−0.346^**^	0.550^**^	−0.342^**^
(PC3) Care-rated change: challenging behaviours	−0.172^*^	−0.524^**^	−0.357^**^	−0.388^**^	−0.335^**^	−0.224	−0.308^**^
(PC4) Impulsive behaviours	−0.006	−0.213^*^	−0.354^**^	−0.428^**^	−0.293^**^	−0.281^*^	−0.397^**^
(PC5) Impulsivity self-report	−0.106	0.041	0.087	−0.03	0.109	0.078	−0.001
(PC6) Goal-directed decision-making	0.055	0.017	0.104	0.037	0.077	0.074	0.023
(PC7) Stop Signal Task	−0.037	0.080	0.172^*^	0.190^*^	0.170^*^	−0.170	0.228^*^
(PC8) Outcome sensitivity	0.032	0.066	−0.029	0.035	−0.057	−0.130	−0.036

^*^P < 0.05; ^**^P < 0.001 (uncorrected, approximating *P* < 0.05 corrected for multiple comparisons).

### Imaging results

**Figure 2:**
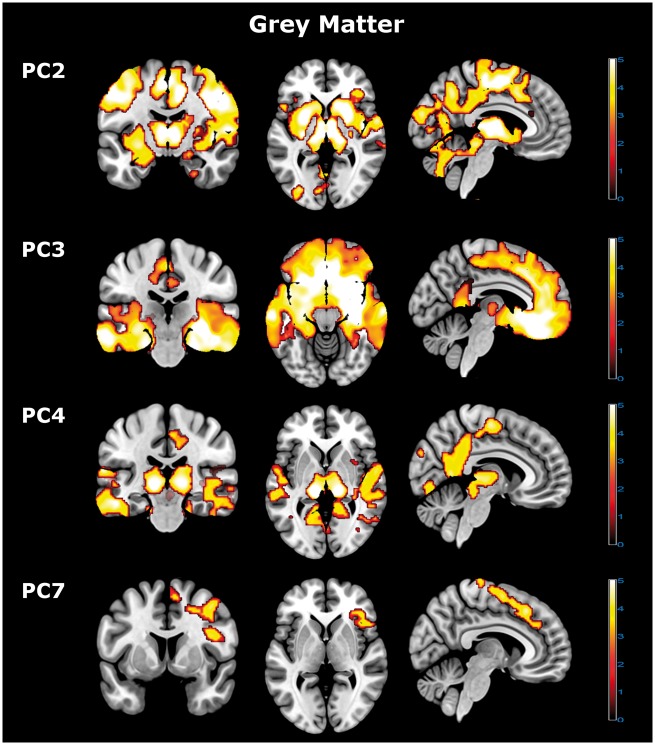
**Grey matter voxel-based morphology imaging results.** Voxel-based morphology analysis revealed distinct neural grey matter correlates for principal Components 2, 3, 4 and 7. Components 2–4 were negative, with higher component scores reflecting a loss of grey matter in the relevant brain regions. Component 7 was positively correlated with the associated brain regions, with higher component scores reflecting increased grey matter in the highlighted areas. Significant effects were identified using cluster-level statistics (FWEc *P* < 0.05, corrected for multiple comparisons) above a height threshold of *P* < 0.005 (uncorrected). PC = principal component.

**Figure 3:**
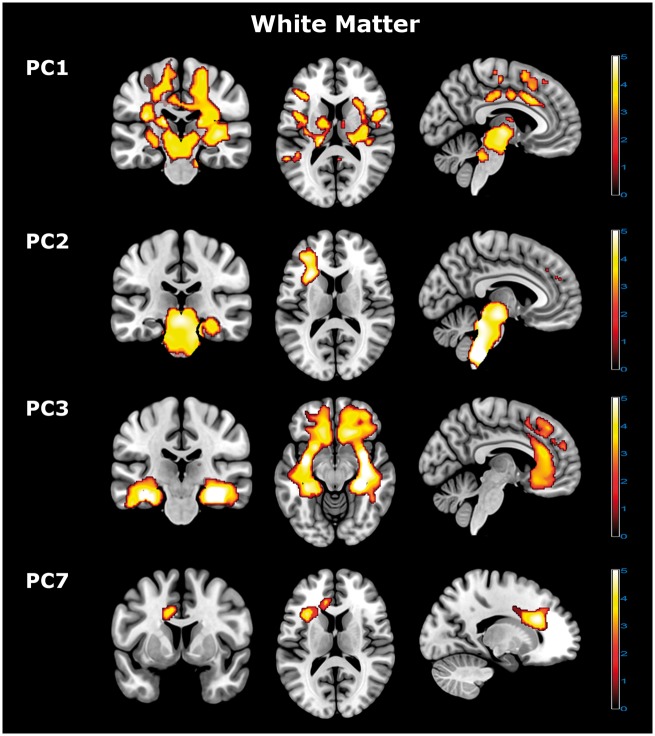
**White matter voxel-based morphology imaging results.** Voxel-based morphology analysis revealed distinct neural white matter correlates for principal Components 1, 2, 3, and 7. Components 1–3 represent negative correlations, with higher component scores reflecting a loss of white matter in the relevant brain regions. Component 7 was positively correlated with the associated brain regions, with higher component scores reflecting increased white matter in the highlighted areas. Significant effects were identified using cluster-level statistics (FWEc *P* < 0.05, corrected for multiple comparisons) above a height threshold of *P* < 0.005 (uncorrected).

The components of apathy and impulsivity were correlated with distinct grey and white matter abnormalities, in corticospinal, frontostriatal and subcortical systems. Figures 2 and 3 illustrate the distributions of significant clusters (multi-slice images for all significant correlations are available as Supplementary material).

Significant white matter correlates were identified for Components 1, 2, 3 and 7 (Fig. 3) and grey matter correlates for Components 2, 3, 4 and 7 (Fig. 2). Note that patients’ (Component 1) and carers’ (Components 2 and 3) ratings were associated with distinct white matter correlates. The patient ratings of Component 1 were related to impairments in the corticospinal tracts, from the mid-centrum semiovale, through corona radiata to the upper brainstem. In contrast, the carer ratings correlated with frontostriatal and brainstem systems. Specifically, carer-rated change in everyday skills and self-care (Component 2) reflected localized brainstem white matter changes (medulla, pons, and lower midbrain largely sparing the thalamus, and white matter deep to the middle frontal gyrus) (Fig. 3 and Supplementary Fig. 4), with grey matter changes extending from the caudate, putamen and thalamus into multiple cortical regions including medial and lateral premotor and sensorimotor cortex, and scattered foci in prefrontal, pariental and occipital cortex (Fig. 2 and Supplementary Fig. 5). Carer-rated behavioural change (Component 3) was associated with widespread but complementary changes in both grey and white matter of the temporal pole, frontal pole, orbitofrontal and medial frontal cortex and their connecting tracts (Figs 2 and 3 and Supplementary Figs 6 and 7).

Performance on the motor/saccade response inhibition, cued reinforcement and information sampling tasks (Component 4) reflected grey matter change in multiple regions including thalamus, lateral temporal cortex, posterior and dorsal-anterior cingulate cortex, and parieto-occipital cortex (Fig. 2 and Supplementary Fig. 8). Performance on the Stop-Signal task (Component 7) reflected localized grey matter change in the right inferior frontal region and anterior cingulate and white matter change in the left frontal lobe (Figs 2, 3 and Supplementary Figs 9 and 10).

## Discussion

Our data provide four critical insights into apathy and impulsivity, in addition to confirming their multifactorial nature. First, apathy and impulsivity are common in all syndromes associated with FTLD, not only those that include apathy and impulsivity as diagnostic criteria. Second, they are positively correlated, such that apathetic individuals are also more likely to be impulsive. Third, the components that reflect patients’ own ratings of apathy and impulsivity are distinct from those based on carer observations and objective behavioural measures. Finally, the anatomical networks associated with apathy and impulsivity in our patients correspond with established networks for goal-directed behaviour, social cognition, motor control and vegetative functions. Specifically, carer ratings (AES, NPI, CBI) reflect widespread disruption in frontostriatal, frontotemporal and brainstem systems required for motivation, goal-directed behaviour and arousal, while patient ratings (AES, BIS, SHAPS, BDI, MEI) correlated with changes in corticospinal tracts, which we suggest reflects patients’ awareness of their motor deficits despite lack of insight into cognitive decline. Objective measures reflected localized changes in previously identified task-specific brain regions (e.g. Stop-Signal task and right inferior frontal gyrus).

In this cross-sectional study, disease progression may have obscured the phenotypic boundaries between syndromes in comparison to their initial presentation (Kertesz *et al.*, 2005; Williams and Lees, 2009). This emphasizes the advantages of transitioning from a traditional ‘nominal’ diagnostic classification (e.g. The Diagnostic and Statistical Manual of Mental Disorders) to dimensional approaches such as Research Domain Criteria with data-driven methods as in our study. The recognition of apathy and impulsivity across syndromes highlights the limitations of diagnostic criteria, and means that symptomatic therapies in one illness may help patients and carers affected by another (Ye *et al.*, 2014, 2015; Hughes *et al.*, 2015). Current criteria do not fully recognize the extent of behavioural changes [e.g. nvPPA (Gorno-Tempini *et al.*, 2011), PSP (Litvan *et al.*, 1996)], or emergence of behavioural disorders with disease progression (e.g. svPPA) (Gorno-Tempini *et al.*, 2011). A clinical trial for such symptoms would be most powerful if stratifying patients into ‘apathetic’ and/or ‘impulsive’ groups across the FTLD spectrum, rather than diagnostic groups, which include patients with and without the relevant symptoms.

This dimensional approach also provides a set of explicit measures of disease severity, in terms of component weights. When combined with the imaging analysis, it enables better characterization of the neural systems underlying behaviours observed across the disorders. The neuroimaging correlates of severity across the different ‘modes’ of apathy and impulsivity provide a principled way to assess the benefits of symptomatic and disease-modifying drugs on the neural systems that regulate different behaviours, and do so using measurement tools that are useful in the context of FTLD syndromes.

Before discussing the individual components, we highlight two general features of the data. First, apathy and impulsivity were positively correlated. This contradicts theoretical models in which impulsivity and apathy represent opposite extremes of a simple spectrum of motivation. Some authors have proposed that impulsivity represents a dopamine-dependent spectrum of motivational or goal-directed control (Zamboni *et al.*, 2008; Ahearn *et al.*, 2012; Sinha *et al.*, 2013) while apathy reflects an independent noradrenaline-dependent spectrum of arousal and uncertainty (Remy *et al.*, 2005; Loued-khenissi and Preuschoff, 2015). However, noradrenaline is also implicated in impulsivity (Ye *et al.*, 2015) and dopamine in apathy (Adam *et al.*, 2013; Sinha *et al.*, 2013), indicating overlapping pharmacology. Although this study did not directly measure or manipulate such neurotransmitters, our results are relevant to the pharmacological analysis of apathy and impulsivity. Specifically, the positive correlation we observe suggests either that there is a common neurobiological basis for apathy and impulsivity (Zhang *et al.*, 2016), or that the widespread pathology in FTLD syndromes leads to simultaneous deficits in anatomically and pharmacologically different networks (Figs 2 and 3).

Second, the cognitive and neural components of apathy and impulsivity differ according to the assessor: patient, carer or experimentalist. The separation of patients’ (Components 1, 5 and 8) and carers’ (Components 2 and 3) ratings likely reflect patients’ lack of insight into disease-related changes or their language difficulty with semantics and grammar in questionnaires. Conversely, carers’ ratings may be biased by their own personal distress (Leroi *et al.*, 2012) or education about the illness. It is unlikely that patients lack insight into all aspects of their disease, but clearly they differ from carers in terms of their awareness of certain symptoms. Eliciting and quantifying behavioural disorders through an interview with carers and/or questionnaires is a feature of both clinical practice and research but may not quantify the differences between a patient’s own symptoms (the usual target of treatment in medicine) and the behavioural signs reported by carers (a major contributor to burden and patient risk). Our findings suggest that clinical trials in syndromes associated with FTLD must distinguish whether treatments are for patients’ or carers’ wellbeing. Furthermore, the subjective questionnaires did not load onto the same components as objective behavioural measures (Components 4, 6 and 7). The identification of homologous tasks in preclinical models and clinical populations can successfully facilitate translational therapeutics (Kehagia *et al.*, 2014; Hughes *et al.*, 2015; Ye *et al.*, 2015), but may not readily apply to FTLD.

Although patients with FTD are said to lack insight, Component 1 correlated with well-defined and largely symmetric neural systems including corticospinal tracts. These correlates differ from atrophy patterns identified from voxel-based morphometry studies of PSP and CBD versus controls (Cordato *et al.*, 2005; Paviour *et al.*, 2006; Josephs *et al.*, 2008*b*; Whitwell and Jack, 2010; Ghosh *et al.*, 2012; Wolpe *et al.*, 2014), which highlight deficits in the medial frontal cortex, parietal lobe and brainstem. We speculate that our result may reflect patients’ awareness of motor deficits, while insight into cognitive decline and behavioural change remains limited.

In contrast to patient ratings, carer ratings of challenging behaviours (Component 3) and vegetative features (Component 2) correlated with frontostriatal and frontotemporal networks for motivational and arousal systems (Chudasama and Robbins, 2006; Rushworth, 2008; Berridge *et al.*, 2010) and brainstem integrity. Both Components 2 and 3 correlated with functional severity (FRS) and cognitive decline (ACE-R, MMSE, FAB), supporting the hypothesized associations between apathy, cognition and functional decline (Starkstein *et al.*, 2006).

Interestingly, both semantic and behavioural variants of FTD were strongly weighted to Component 3. Although svPPA is primarily diagnosed as a language disorder with temporal lobe atrophy, the spread of pathology to orbitofrontal systems and increasing behavioural change indicate partial convergence of svPPA and bvFTD phenotypes (Hodges and Patterson, 2007). The neural correlates of Component 3 (Figs 2 and 3) suggest disrupted motivation and reward processing circuitry with both apathy and impulsivity, consistent with the regulation of reward, motivation and reinforcement by projections from the orbitomedial prefrontal cortex and anterior cingulate to ventral striatum (Dalley *et al.*, 2011; Ahearn *et al.*, 2012; Eslinger *et al.*, 2013). Carer ratings closely reflect changes in these brain circuits previously implicated in apathetic and impulsive behaviours (Levy and Dubois, 2006; Zamboni *et al.*, 2008; Sinha *et al.*, 2013). Analogous changes have been observed in many neurological and psychiatric impulsivity disorders (Levy and Dubois, 2006; Dalley *et al.*, 2011; Ersche *et al.*, 2011; Sinha *et al.*, 2013).

The white matter correlates of Component 2 (everyday skills and vegetative functions) were concentrated in the brainstem (Fig. 3), with grey matter correlates extending from the thalamus to posterior regions of cingulate and parietal cortex (Fig. 2). These changes were most strongly associated with PSP and CBS, consistent with previous reports (Burrell *et al.*, 2014). Degeneration of the brainstem is proposed to affect the reticular activating system that regulates wakefulness, attention and alertness. Furthermore, sustained attention and oculomotor control require functional integration of the brainstem, thalamus and neocortical areas associated with this component, and are particularly affected by PSP and CBS.

In other neuropsychiatric studies of impulsivity, including addiction and Attention Deficit Disorder, the BIS and BIS/BAS questionnaires have been used to quantify individual differences. These tests loaded onto Component 5. Similar questions partly explain their presence in the same component (e.g. BIS/BAS: ‘I often act on impulse’ versus BIS: ‘I act on impulse’). But, the transdiagnostic plots (Fig. 1) suggest that such responses do not readily distinguish patients affected by FTLD.

The stop-signal task was weighted to Component 7. Previous studies of health, Parkinson’s disease, ADHD and ageing have consistently associated this task with the integrity, activity and connectivity of the right inferior frontal gyrus (Aron *et al.*, 2003; Dalley *et al.*, 2011), presupplementary area and subthalamic nucleus (Aron *et al.*, 2004), as well as noradrenergic (Kehagia *et al.*, 2014; Ye *et al.*, 2015) and serotoninergic (Ye *et al.*, 2014) function. Higher scores on Component 7 correlated with increased grey matter volumes in the right inferior frontal gyrus and its connections to the striatum, providing further construct validation of our dimensional approach.

The last and weakest component we termed ‘outcome sensitivity’ due to its loadings from the Kirby and BIS/BAS’s BIS subscore. The BIS subscore reflects a system for relaying cues of punishment, non-reward and novelty, to regulate behaviour (Amodio *et al.*, 2008). In the Kirby paradigm, steeper discounting has been reported in drug addiction, schizophrenia and Parkinson’s disease (Housden *et al.*, 2010). Group comparisons (Fig. 1) and the lack of significant anatomical correlates are consistent with this component being a trait in the general population, rather than a disease-specific deficit.

Our study has methodological and interpretative limitations. Although we aimed to assess the multifaceted constructs of apathy and impulsivity, some patients could not perform the tasks, and the Cambridge Gambling Task proved especially difficult despite its successful application in milder neuropsychiatric populations. The task was withdrawn after 37 participants, but inclusion of these additional data did not alter the factor structure significantly, and although patients were poor on the task, this effect was captured by other tasks including the cued reinforcement reaction time task (Cools *et al.*, 2005). Interestingly, pathological gambling is uncommon even in bvFTD, and the impairment may arise partly from executive deficits.

Some of the assessment tools were disease-specific, or developed for a particular cohort, limiting their generalization. For example, the FRS may not be directly applicable to PSP and CBS. It could be therefore argued that one should assess the neural correlates of performance separately within each diagnosis. However, reducing the analysis to a multiplicity of tests of individual symptoms within syndromes would have significant drawbacks, not just in terms of the severe loss of power to detect correlations in small sub-cohorts. It would also belie the evidence of clinical overlap and convergent symptomatology across the separate diagnostic groups. Moreover, the use of factor loadings for each component for each patient provides a more principled means to accommodate syndromic variance, without bias or diagnostic circularity.

We sought to obtain the maximum information about potential aspects of apathy and impulsivity, whilst bearing in mind the tolerance and frailty of patients with FTLD-associated disorders. However, our test battery is selective and our conclusions only relate to the domains of cognition and behaviour assessed. Some tasks that quantify apathy in the healthy population are especially challenging in FTLD disorders, because of sequential decisions, physical effort and strong executive demands. For example, grip-force effort (Bonnelle *et al.*, 2015; Chong *et al.*, 2015; Bouc *et al.*, 2016) might be confounded by the movement disorders in several FTLD syndromes. Akinesia, depression and executive deficits may confound the assessment of apathy.

Akinesia may readily be confused with apathy by observers. However, we suggest it is unlikely that the apathy we identify is driven solely by akinesia, as akinesia across diagnostic groups does not mirror the severity of apathy (Table 3 and Fig. 1). We indirectly measured motor features, in terms of physical signs (including akinesia in the PSPRS) and as reaction times in objective behavioural tests. The correlations between the principal components and PSPRS were very limited (Table 5). Depression can also confound the assessment of apathy. Indeed, patient-rated apathy, depression and anhedonia scores were positively correlated (Component 1), despite distinctions between the proposed underlying neurobiology of these complications (Levy *et al.*, 1998). However, self-rated depression symptom scores, as measured the BDI-II, are distinct to the clinical disorder of depression that is primarily a mood disorder. Apathy and depression may have common symptoms, and both contribute to high scores on a questionnaire such as the BDI-II, even as distinct pathological entities. The role of executive function in task performance must also be considered. Executive deficits are part of the diagnostic criteria for bvFTD, and supportive criteria for PSP, and yet they are common in other disorders associated with FTLD (Burrell *et al.*, 2014). However, a simple deficit in executive function cannot account for the fractionation of apathy and impulsivity as revealed by the PCA, nor the separate neural correlates of each component. Rather, the separate impairments in behavioural control, inhibition, goal-directed behaviour and appropriate planning of responses can be construed as a part of the complex dysexecutive status resulting from FTLD. Indeed, verbal fluency, a marker of executive function (Perneczky *et al.*, 2011), correlated with Components 1–4 and 7, in keeping with the association between executive functions and frontal lobe function (Shallice, 1988). We suggest that executive dysfunction in our cohort is best seen as encompassing—but not causing—the components of apathy and impulsivity we observe.

Voxel-based morphology changes in white matter should be interpreted with caution (Smith *et al.*, 2006), especially where white matter correlates are observed in the absence of grey matter correlates (e.g. Component 1). They may reflect true white matter influences on complex behavioural repertoires, but false positive correlations may arise from normalization and mislocalization errors and the partial-volume effects of smoothing. In contrast, the complementarity of white and grey matter correlates of Components 2 and 3 strengthens their interpretation. Voxel-based morphology has been used extensively in the literature to examine white matter volumes in PSP (Brenneis *et al.*, 2004; Cordato *et al.*, 2005; Boxer *et al.*, 2006; Josephs *et al.*, 2008*b*; Ghosh *et al.*, 2012; Dutt *et al.*, 2016), CBS/D (Boxer *et al.*, 2006; Josephs *et al.*, 2008*b*; Whitwell and Jack, 2010; Dutt *et al.*, 2016) and FTD (Rohrer *et al.*, 2011). However, alternative methods are increasingly common to study white matter changes in FTLD syndromes, including diffusion-weighted imaging with voxel-wise regions of interest or tract-based statistics (Whitwell *et al.*, 2010; Mahoney *et al.*, 2014; Mandelli *et al.*, 2014). Despite differences in assumptions, confounds and sensitivity, there is generally consensus across these methods and voxel-based morphology in the regional effects of FTLD syndromes on white matter.

It is possible that our cohort is biased or unrepresentative of the full spectrum of disorders associated with FTLD. However, the study used multiple sources of referral in community and specialist services, to reach all regional patients, and our attrition from case identification (*n* = 204) to neuropsychological assessment (*n* = 149) and MRI (*n* = 70) included all disorders, while the imaged subset was representative of the whole neuropsychological cohort. Finally, we rely on clinicopathological correlations and the current consensus criteria, acknowledging that for some disorders (nvPPA, CBS and bvFTD) the clinicopathological correlations are weaker than others (svPPA, PSP).

In conclusion, we report that apathy and impulsivity are common and overlapping consequences of FTLD. Structural brain imaging revealed corticospinal tract impairments in relation to patient ratings, in contrast to carer ratings, which correlated with frontostriatal, frontotemporal and brainstem systems. Objective tasks and subjective questionnaires used to measure these multifaceted constructs do not correlate, warranting improved clinical assessment tools to facilitate clinical trials. We argue that a dimensional approach to investigate complex behavioural changes is necessary and provides new insights into apathy and impulsivity as well as refining targets for novel drug treatments.

## Funding

This work was primarily funded by the NIHR Cambridge Biomedical Research Centre with additional support from the Cambridge Home and EU Scholarship Scheme, the James F McDonnell Foundation (21st Century Science Initiative for Understanding Human Cognition), Wellcome Trust (103838); Medical Research Council (MC US A060 0016, and RG62761), the Cambridge Brain Bank, PSP Association and the Evelyn Trust. The BCNI is supported by a joint award from the Wellcome Trust and Medical Research Council. We thank the PSP association & FTD Support Group for raising awareness of the study.

## Conflicts of interest

Trevor W. Robbins: Consultancy for Cambridge Cognition, Lundbeck and Otsuka. Research grants from Lundbeck and GSK. Royalties for CANTAB from Cambridge Cognition. Editorial honoraria from Psychopharmacology (springer) and Current Opinion in the Behavioural Sciences (Elsevier). James B Rowe: Research Grant from AZ Medimmune.

## Supplementary material

Supplementary material is available at *Brain* online.

## Supplementary Material

Supplementary Data

## Abbreviations

ACE-R: Addenbrooke’s Cognitive Examination-Revised
BIS: Barratt Impulsiveness Scale
bvFTD: behavioural variant frontotemporal dementia
CBS: corticobasal syndrome
FRS: Frontotemporal Dementia Rating Scale
FTLD: frontotemporal lobar degeneration
PPA: primary progressive aphasia
PSP: progressive supranuclear palsy
