# Supplementary material for: Apathy and impulsivity in frontotemporal lobar degeneration syndromes
Source: Brain. 2017 May 9;140(6):1792–807. doi: 10.1093/brain/awx101 (PMC5868210; doi:10.1093/brain/awx101)

SUPPLEMENTARY MATERIAL

Figure 1: Diagram of PCA method.

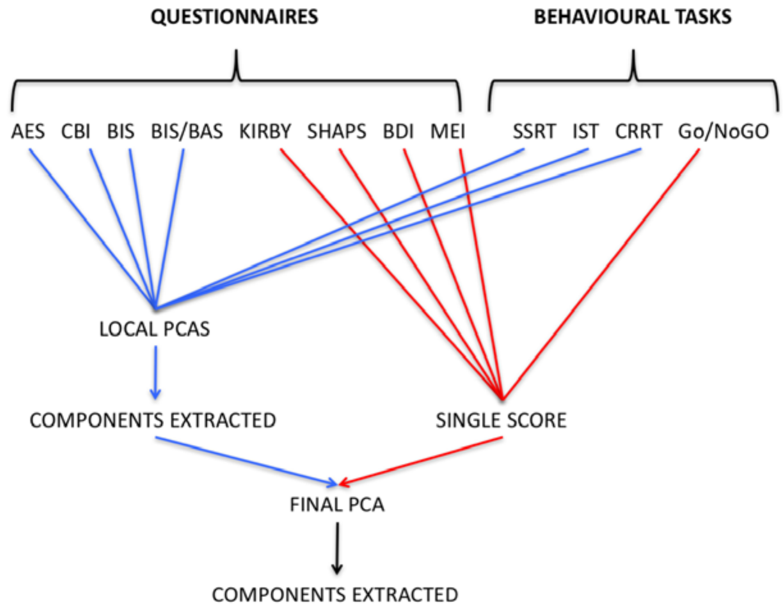

Local PCAs were carried out on all questionnaires and behavioral tasks independently, where appropriate. Components were then extracted from a final PCA. Note: the Go/NoGO task was run as both a motor and saccade task (2 variables).

Table 1: Comparison of Imaging Subset (N=70) and Non-Imaging Subset (N=79) of the PCA Sample (N=149)

	Variable	Imaged (N=70)	Non Imaged (N=79)	T Stat	P value (unc)
**Demographics & Cognition**	Age	68.2±8.2	70.4±8.6	1.6	0.12
	Gender M:F	39:31	37:42	(χ^2=1.2)	0.33
	ACE-R Total (max 100)	67.3±22.2	58.9±23.0	-1.7	0.10
	MMSE Total (max 30)	23.0±6.8	20.7±6.7	-1.6	0.12
	FRS % Score (max 100)	41.4±27.9	32.5±23.6	-1.8	0.08
**Questionnaires**	Apathy Evaluation Scale (AES, max 72):				
	-carer	46.5±12.6	50.8±11.7	1.8	0.08
	-patient	36.6±9.2	34.5±10.2	-0.8	0.44
	-clinician	43.1±9.6	45.1±11.3	0.76	0.45
	Barratt Impulsivity Scale (BIS, max 120)	64.1±7.9	61.5±8.8	-1.1	0.29
	Behavioural Inhibition System/Behavioural Activation System (BIS/BAS):				
	-BIS subscore	20.8±4.7	19.9±3.5	-0.6	0.54
	-BAS drive	11.0±3.3	10.8±2.9	-0.2	0.83
	-BAS funseeking	11.2±2.9	11.7±3.8	0.5	0.60
	-BAS Reward Responsivness	16.4±2.7	17.4±2.8	1.2	0.25
	Motivation and energy inventory (MEI, max 144)	80.5±27.2	83.8±22.6	0.4	0.69
	Beck depression inventory (BDI, max 63)	13.1±10.7	12.7±7.6	-0.2	0.88
	Snaith Hamilton pleasure scale (SHAPS, max 56)	22.3±5.1	23.1±3.8	0.6	0.58
	Neuropsychiatric inventory (NPI, fraction with positive response):				
	-Apathy subscore	.59±.50	.66±.48	0.7	0.46
	-Disinhibition subscore	.35±.50	.31±.47	-0.5	0.65
	Cambridge behavioural inventory (CBI-R, max 180)	62.0±35.7	73.8±33.7	1.8	0.08
	Kirby (difference)	.01±.05	.04±.07	1.5	0.13
**Behavioural Tasks**	Information Sampling Task (IST)				
	-Probability of being correct Fixed	.75±.15	.71±.11	-0.8	0.41
	-Probability of being correct Decreasing	.67±.17	.66±.08	-0.1	0.89
	Cued reinforcement reaction time (CRRT)				
	-reward related speeding	62.1±331.8	128.0±853.7	0.57	0.57
	-Total Errors	4.1±5.0	4.5±8.3	0.2	0.82
	Stop Signal Task (SST)				
	-Stop signal reaction time (SSRT)	401.8±213.1	430.6±242.3	0.4	0.81
	Motor Go/NoGO Dprime	3.2±1.3	3.0±1.2	-0.7	0.48
	Saccade Dprime	.79±1.1	.62±1.1	-0.4	0.68

Table 2: Summary of Patient Characteristics by Diagnostic Group

	Variable	Controls	Patients (all groups)	PSP	CBS	bvFTD	PPA
	N	50	149	41	37	32	39
Demographics	Age	70.6 ±6.5	69.3 ± 8.5	72.1±8.3	69.4±8.2	63.9±8.0	71.0±7.3
	Gender M:F	23:27	76:73	21:20	18:19	18:14	19:20
	ACE-RT	95.6 ±4.4	64.7 ± 22.6	75.5±14.6	65.7±21.3	59.0±27.0	54.8±23.6

otal (/100)						
MMSE Total (/30)	29.3 ±1.2	22.3 ± 6.8	25.0±4.8	22.0±6.6	21.4±7.6	19.9±7.8
FRS% Scor	92.1 ±10.8	37.9 ± 26.5	40.9±25.1	31.4±23.3	23.4±6.0	50.1±31.8

	e (/1000)						
Questionnaires	Apathy Evaluation Scale (AES	24.2 ±5.7	48.1 ±12.4	48.4±10.9	48.6±11.2	54.3±9.4	42.6±14.9
		25.7 ±5.6	36.1 ±9.4	39.1±11.2	36.1±6.8	32.3±9.6	35.2±7.8
		25.9 ±7.3	43.6 ±10.0	47.1±11.0	45.2±8.2	43.2±7.3	36.9±9.9

/72): *-carer-patient-clinician*						
Barr	57.0 ±7.4	63.6 ±8.1	65.2±7.3	61.8±10.2	63.3±6.7	63.2±8.0

att Impulsivity Scale (BIS/120)

Behavioral Inhibition System Behav	19.9 ±3.4	20.6 ±4.5	19.8±3.2	21.9±3.3	19.3±3.3	21.8±7.0
	10.0 ±2.1	10.9 ±3.2	11.0±3.1	9.9±3.4	12.6±3.2	10.5±7.0
	10.7 ±2.2	11.3 ±3.0	10.7±2.8	10.3±3.7	12.7±3.2	11.8±2.1
	15.8 ±2.4	16.6 ±2.7	16.2±2.7	17.3±2.2	16.7±3.6	16.5±2.2

ioral Activation System (BIS/BAS): - *B*

ISsubscore-BASdrive-BASfunseeki

ng-BAS Reward Responsiveness						
Motiva	108.9 ±17.2	81.1 ±26.4	70.7±29.5	74.1±23.5	96.9±23.9	90.3±15.9

tion and energy inventory (MEI/144)

Beck depression inventory (BDI/6	4.2 ±4.0	13.0 ±10.1	17.8±11.5	14.3±8.0	9.3±6.1	7.8±9.4

3)						
Snaith Hamilton pleasure scale (SH	18.6 ±4.4	22.5 ±4.8	22.3±4.3	24.1±5.1	26.8±18.0	20.25±3.5

APS/56)						
Neuropsychiatric inventor	.000 ±.0<br>.020 ±.1	.62 ±.5<br>.33 ±.5	.63±.5<br>.23±.4	.78±.4<br>.19±.4	.68±.5<br>.56±.5	.40±.5<br>.40±.5

y (NPI, fraction with positive response):

- Apathy subscore - Disinhibition su

bscore						
Cambridge behavioral invent	5.2 ±5.6	66.7 ±35.2	56.0±32.4	73.5±30.6	85.6±26.2	55.7±41.1

	ory (CBI-R/180)						
	Kirby (difference)	.005 ±.04	.019 ±.1	.035±.06	.017±.04	-.00018±.08	.008±.03
**Be**	In	.866 ±.1	.747 ±.1	.743±.1	.705±.1	.846±.1	.706±.1

f<br>o<br>r<br>m<br>a<br>ti<br>o<br>n<br>S<br>a<br>m<br>p<br>li<br>n<br>g<br>T<br>a<br>s<br>k<br>(I<br>S<br>T<br>)<br>-<br>P<br>r<br>o<br>b<br>a<br>b	.806 ±.1	.668 ±.2	.678±.1	.617±.2	.721±.2	.654±.1

ility of being correct Fixed-Probabil

ityofbeingcorrectDecreasing						
Cued	-43.4±90.9<br>3.8±3.4	196.3±739.1<br>4.2±5.7	31.6±581.3<br>3.8±3.2	183.4±235.1<br>4.0±4.2	-1.81±305.9<br>2.4±2.1	657.8±1230.9<br>6.8±10.3

reinforcement reaction time (CRRT)

-DifferenceSpeeding-Totalerrors

Stop Signal Task (SST)-Stop signal	181.1 ± 41.7	439.8±190.4	431.7±146.1	435.2±189.3	367.4±154.7	512.6±251.5

reaction time (SSRT)						
Motor Go/NoGO Dp	4.4 ±.3	3.2±1.3	3.3±1.1	2.8±1.4	4.0±1.5	3.1±1.4

ri me						
Saccade D prime	2.4 ±.9	.75±1.1	.71±.9	.98±1.2	1.1±1.4	.4±1.0

Demographics and disease characteristics by diagnostic group, split into equally weighted groups of PSP, CBS, bvFTD and PPA. Note that PPA included 12 nvPPA, 11 svPPA and 11 “PPA other” cases (2 lvPPA and the remaining not meeting criteria for either svPPA or nvPPA and therefore unspecified).

A Detailed Description of Behavioural Tasks

Commented [CL1]: More detail needed here

Motor and Saccadic Go/NoGO Task

The Go/NoGO task is a measure of response inhibition, specifically action restraint, which is distinct from action cancellation measured by the stop signal task. The saccade Go/NoGO task is explained in detail in the main text. The motor NoGO task was analogous to the saccadic task but used a joystick operated by the dominant hand (except where physical disability impaired hand use, in which case the most physically able hand was used). Stimuli were presented on a laptop screen positioned 1m from the subject, with the initial red and green cues presented in the top center of the screen. Subjects were instructed to initiate movement (Go trials, green central cue remaining) or inhibit movement (NoGO trials, red central cue remaining) in the direction of the presented arrow pointing left or right. Outcome measures for saccadic and motor NoGo tasks were identical to facilitate direct comparisons and included average reaction times for each trial type, commission and omission errors.

Specifically, variables included: Go Correct Right Direction, Go Incorrect Wrong Direction, NoGo Correct, NoGo Incorrect. Mean reaction times (in milliseconds) for each of the Go responses and the mean number of each response type were calculated. Outliers greater than three standard deviations from the mean (within patient or control group) were excluded from each outcome variable. To provide a measure of performance accuracy, the sensitivity index or dprime was calculated (d'), representing the difference between the correct "hit" rate and incorrect "false-alarm" rate. The hit rate reflected the proportion of Go trials to which the subject correctly responded Go (in either direction) and the false alarm rate reflected the portion of all NoGO trials whereby the patient incorrectly responded GO. Higher d' reflected improved performance.

Cued Reinforcement Reaction Time Task (CRRT)

The CRRT is an assessment of incentive motivation, designed to assess responsiveness to reward signals on an odd one out task (Cools et al., 2005). The task was simplified from the original CRRT to 2 colour/probability options instead of 3 and 50 trials instead of 100. The task was run on a laptop with responses recorded by a 3-button box (dominant hand). Forty

practice trials without feedback were used to familiarize the participant with the task and to titrate reaction time thresholds for each individual (to ensure motivationally relevant signals were tailored to individual differences in cognitive speed) using a cut-off value for reward feedback of mean reaction time minus one standard deviation. Participants were presented with a cue (coloured rectangle), signaling the probability of reward following a correct response, either 20% or 80%. Participants were informed that the chance of receiving feedback was dependent on the colour of the box surrounding the presented circles, but were not informed which colour was more likely to give feedback. Participants then identified the ‘odd-one-out’ of three presented circles. Feedback was; 100 points for a correct and fast response, 1 point for a correct but slow response and 0 points for an incorrect response. Participants were instructed to obtain as many points as possible, for which normal controls demonstrate a “reinforcement-related speeding” effect: responding quicker under the anticipation of increased probability of reward (Cools et al., 2005; G. K. Murray et al., 2008). In order to assess the impact of learning, we examined the mean RT at both reward probability values for the first half (FH) and second half (SH) of trials and then calculated the reward-related speeding effect (eg. First half mean RT 20% probability – First half mean RT 80% probability). We subsequently calculated the overall reinforcement related speeding (Speeding SH – Speeding FH). We further examined the impact of feedback on speed of response to the subsequent trial, providing a measure of response to positive and negative stimuli (positive feedback mean RT and negative feedback mean RT calculated). Additional outcome variables included total errors and total score.

Information Sampling Task (IST)

The Cambridge Neuropsychological Test Automated Battery (CANTAB) IST (Clark et al., 2006) was administered on a touch screen computer to assess reflection impulsivity using five fixed condition and five descending win condition trials. Participants were presented with a 5x5 matrix of 25 grey boxes which, when selected, turned blue/yellow. On fixed trials, participants were instructed to open as many boxes as they liked, before deciding whether there were mostly blue or yellow boxes. On decreasing trials, every selected box subtracted 10 points from a starting sum of 250, to encourage faster decision making based on limited information. Correct responses were rewarded with 100 points and incorrect were punished -100 points. Outcome measures were determined for fixed and decreasing win trials (Cambridge Cognition Ltd.) and included the probability of being correct at the time of making the decision (pCorrect), mean box opening latency (boxLatency, mean colour decision latency (colourLatency), mean boxes opened per trial (boxesOpened), incorrect decisions based on

insufficient evidence (sampling error), incorrect decision based on available evidence (discrimination errors) and total correct decisions (tCorrect).

Stop Signal Task (SST)

The SST is a response inhibition (impulse control) task focusing specifically on action cancellation (Chamberlain et al., 2007). The SST was administered using the CANTAB and a two button press pad. In the first practice part (16 trials), stimuli were presented on a computer screen and participants were instructed to press the right/left button as quickly as possible in response to the right/left arrow. The second part consisted of 64 trials, by which participants were instructed to continue responding as quickly as possible, but to refrain from responding when they heard an auditory signal (beep), presented in 25% of trials (randomly dispersed). The delay between presentation of the arrow stimuli and the stop signal varied, known as the stop signal delay), in order to give an estimate of the stop signal reaction time (SSRT; the time it takes to successfully inhibit a response). The SSRT, the primary outcome measure, is calculated by subtracting the mean SSD at which a participant is able to inhibit responding on 50% of trials (SSD 50) from the mean reaction time on go trials (no stop signal). Following successful inhibition, SSD decreased and when participants failed to inhibit responding, SSD increased. The major outcome variables included SSD, SSRT, total correct responses on stop and go trials, direction errors on stop and go trials, mean/median reaction times for all go trials.

Cambridge Gambling Task (CGT)

The CGT is a unique task assessing decision-making and risk taking behavior in the absence of learning or information retrieval. It dissociates risk taking from impulsivity, as participants have to wait for a risky bet in the ascending condition (Manes et al., 2002). The task was administered using the CANTAB and consisted of a neutral part and two gambling parts. Participants were presented with a row of red and blue boxes at the top of the screen, and were instructed to guess which colour box a yellow token was placed under. Participants responded by touching the boxes containing the words ‘Red’ or ‘Blue’ at the bottom of the screen.

In the gambling stages, participants started with 250 points and could select how confident they are with their decision by gabling a certain proportion of these points, which were displayed on the right hand side of the screen in either ascending (part 1) or descending (part 2) order.

Participants were instructed to obtain as many points as possible, and the total accumulated points is displayed on the screen throughout.

Note: The CGT was removed from the protocol after 37 participants due to floor effects and difficult task engagement by FTLD patients, even following simplification of the task. This highlights the need to develop a disease-specific task to look at gambling behaviours in FTLD syndromes.

Multi Slice Images

Below are the multi-slice figures for all principal components with significant grey and white matter correlates (threshold p<.005, FWEc p<.05). These include white matter correlates for Components 1-3 and 7 and grey matter correlates for Components 2- 4 and 7.

Figure 2: Axial, Coronal and Sagittal Slice Numbers

Axial Slice Numbers

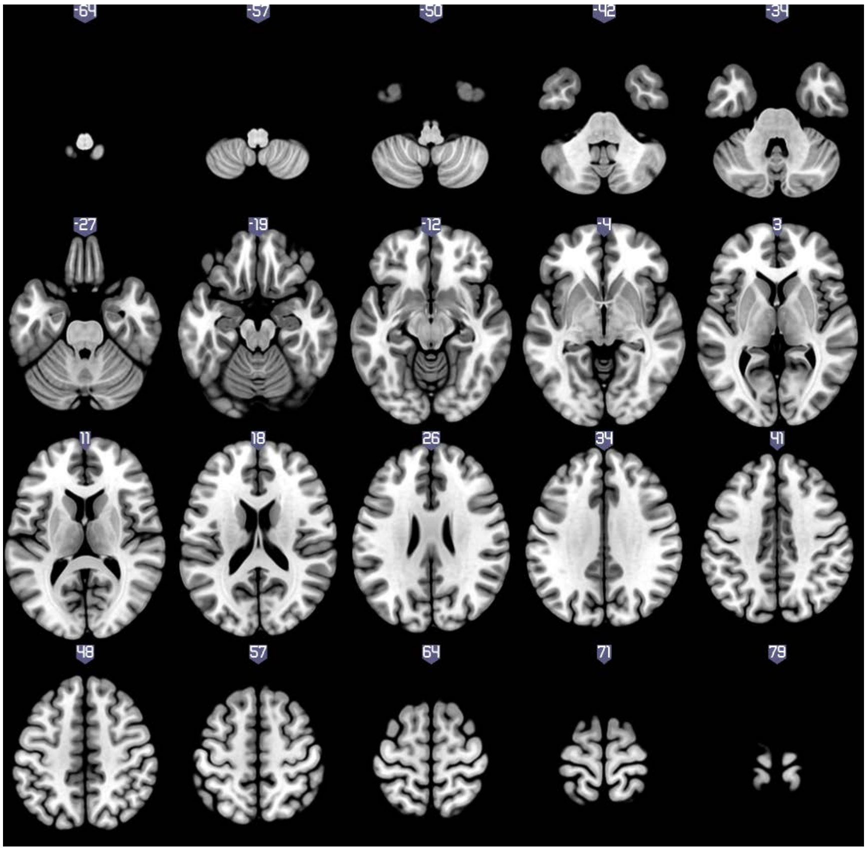

<u>Coronal Slice Numbers</u>

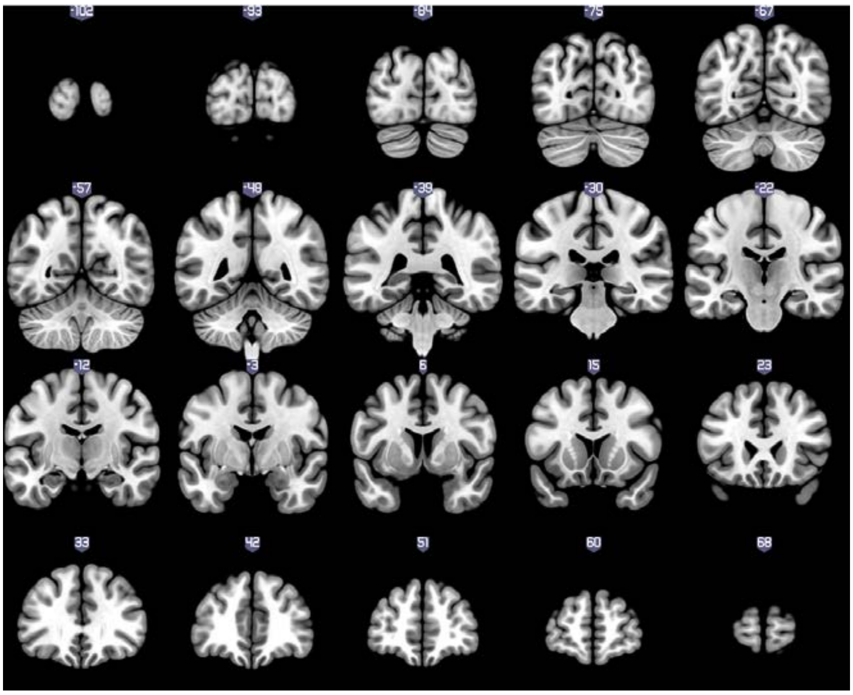

<u>Sagittal Slice Numbers</u>

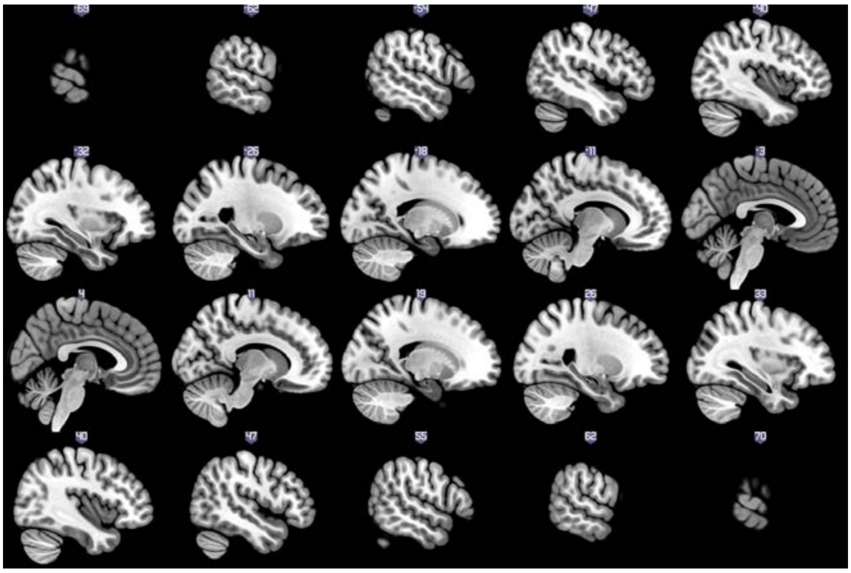

Figure 3: White Matter Correlates of Patient Rated Change (Component 1-ve)

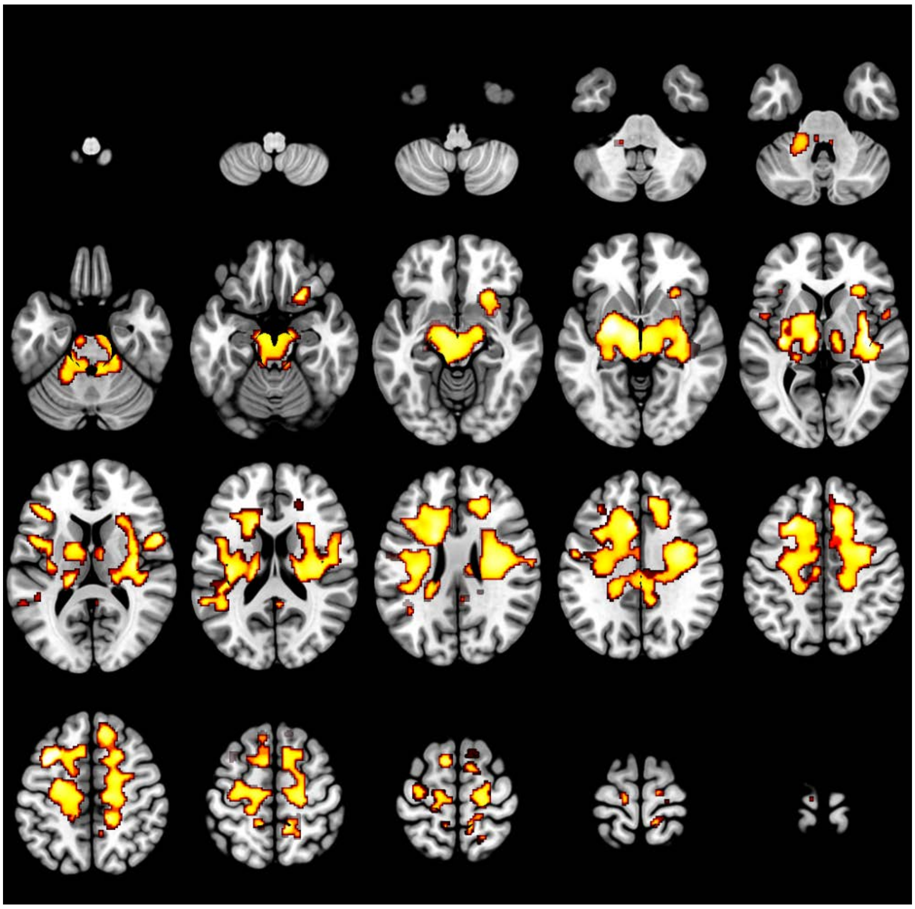

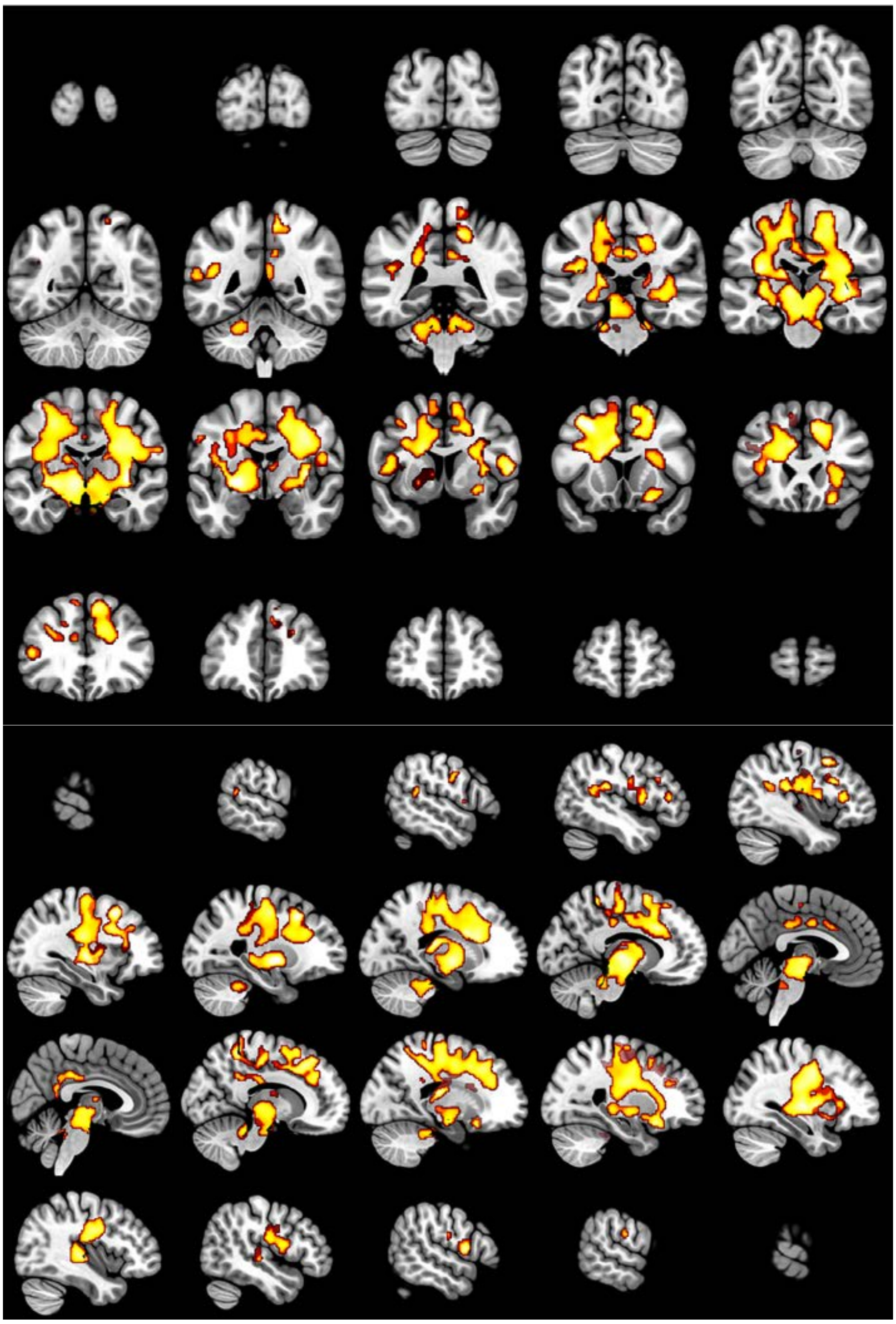

<u>Figure 4: White Matter Correlates of Carer Rated Change in Everyday Skills and Self Care (Component 2-ve)</u>

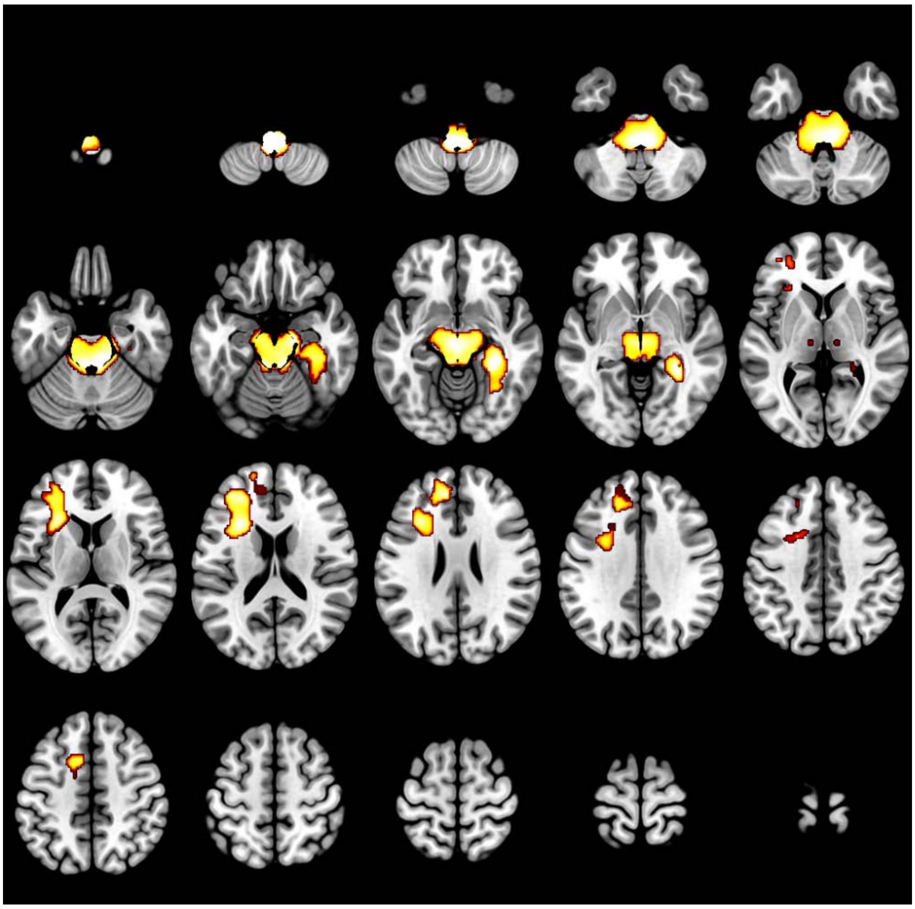

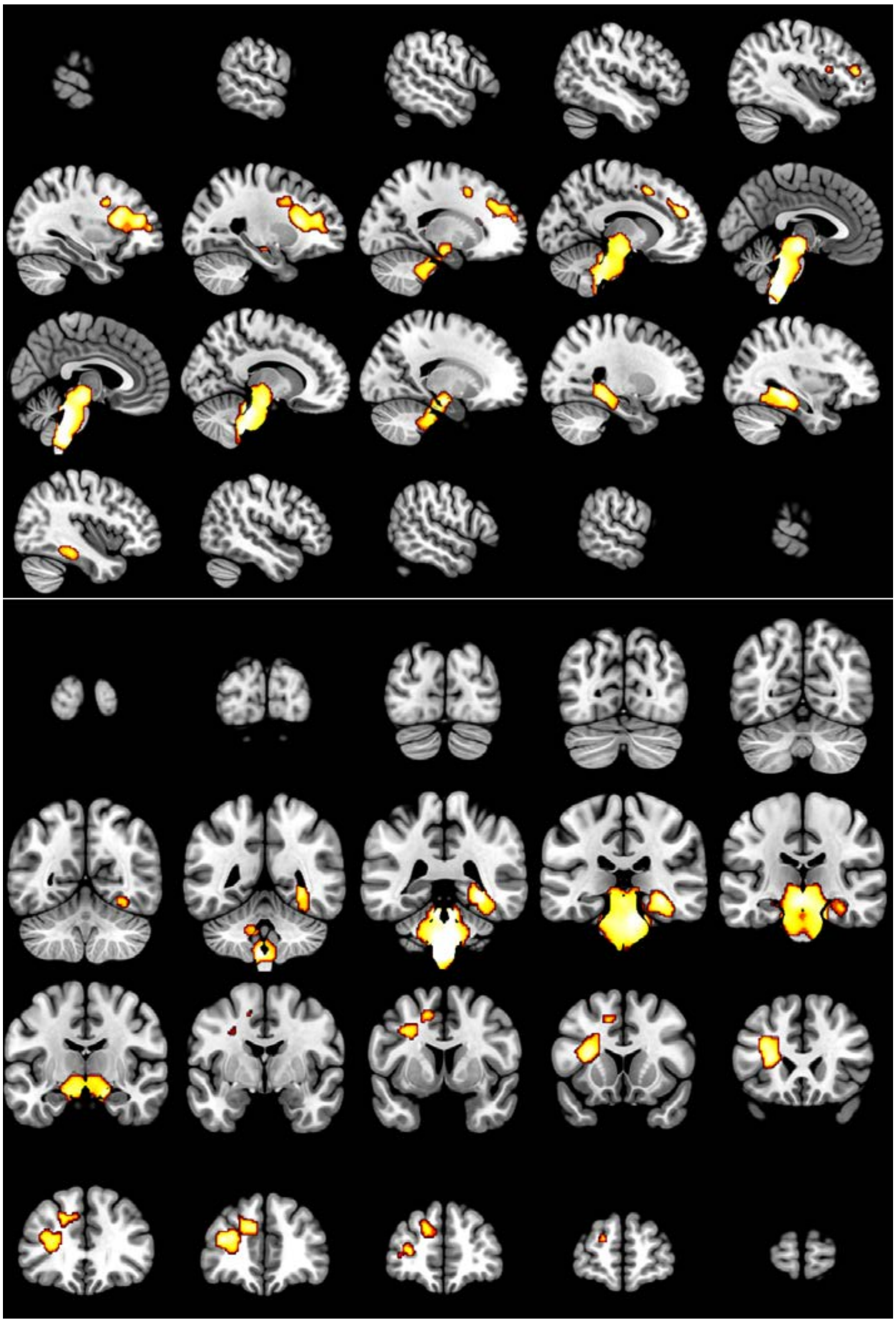

Figure 5: Grey Matter Correlates of Carer Rated Change in Everyday Skills and Self Care (Component 2-ve)

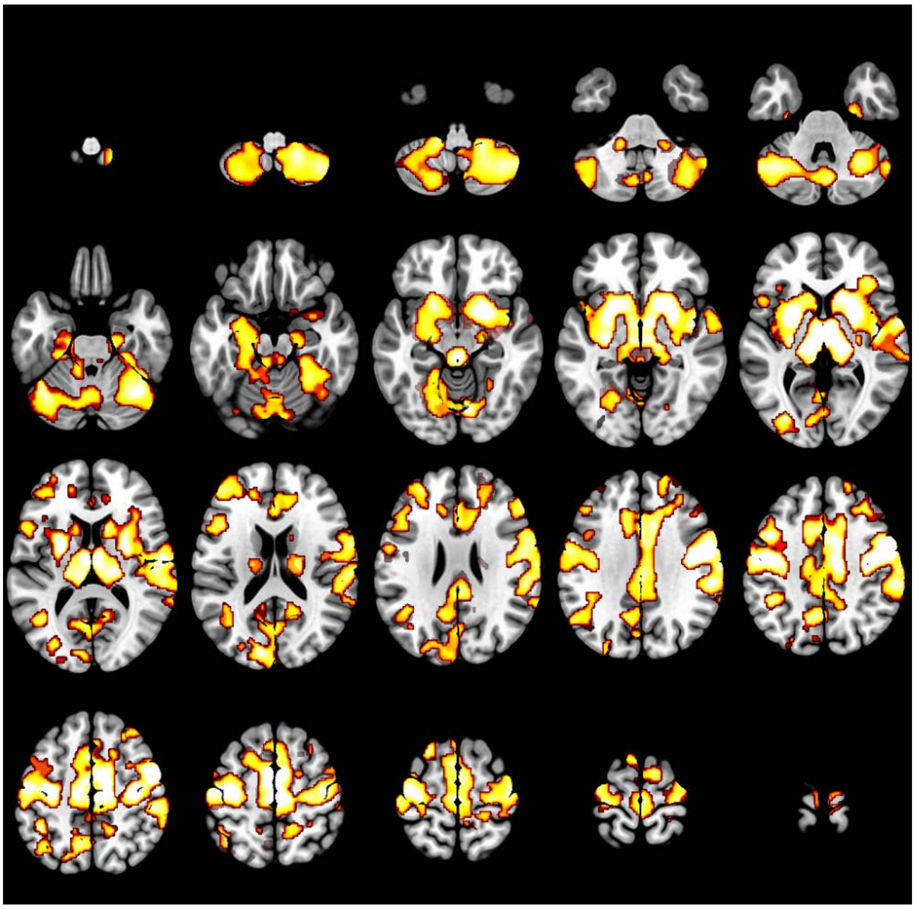

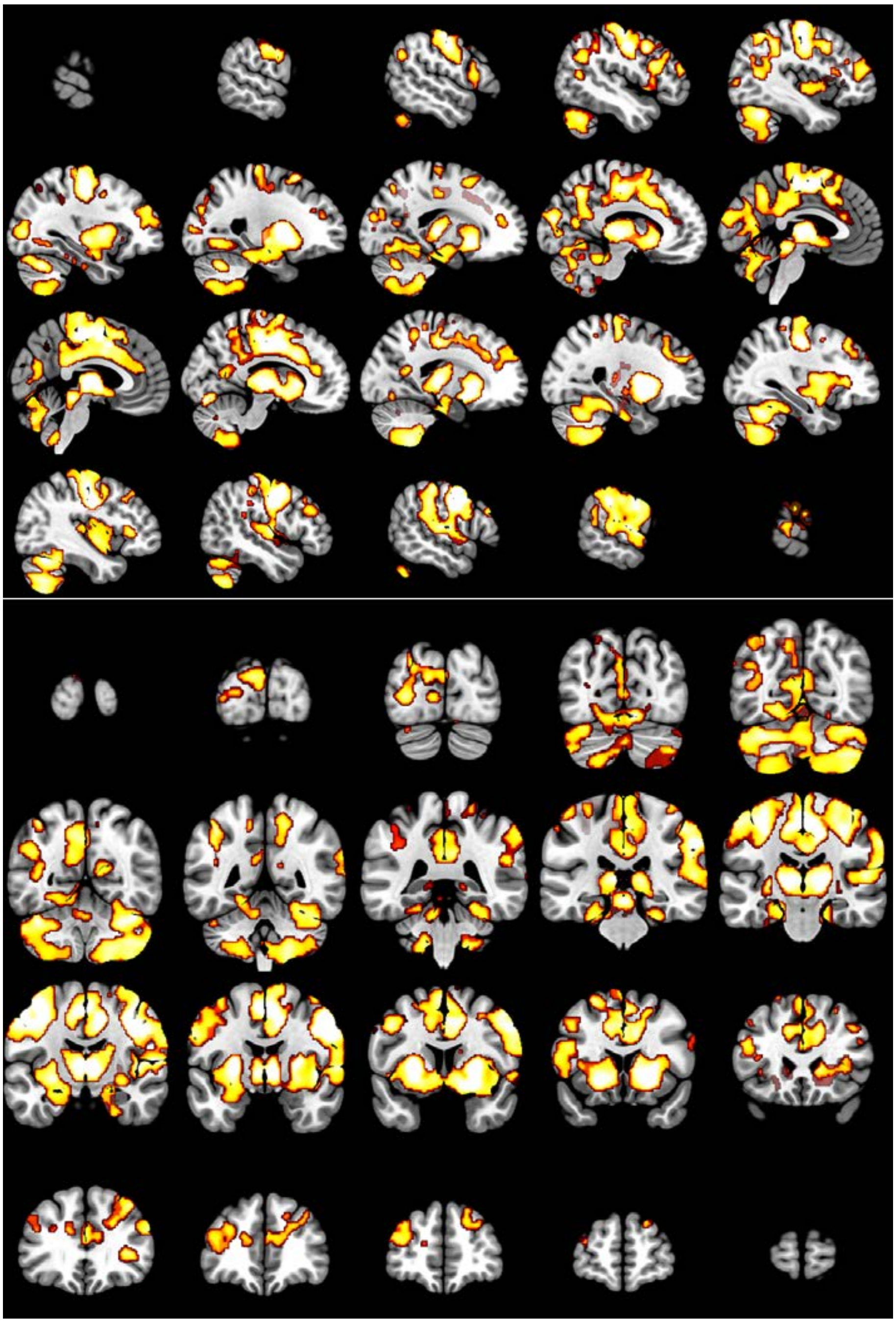

Figure 6: White Matter Correlates of Carer Rated Change Behaviour (Component 3-ve)

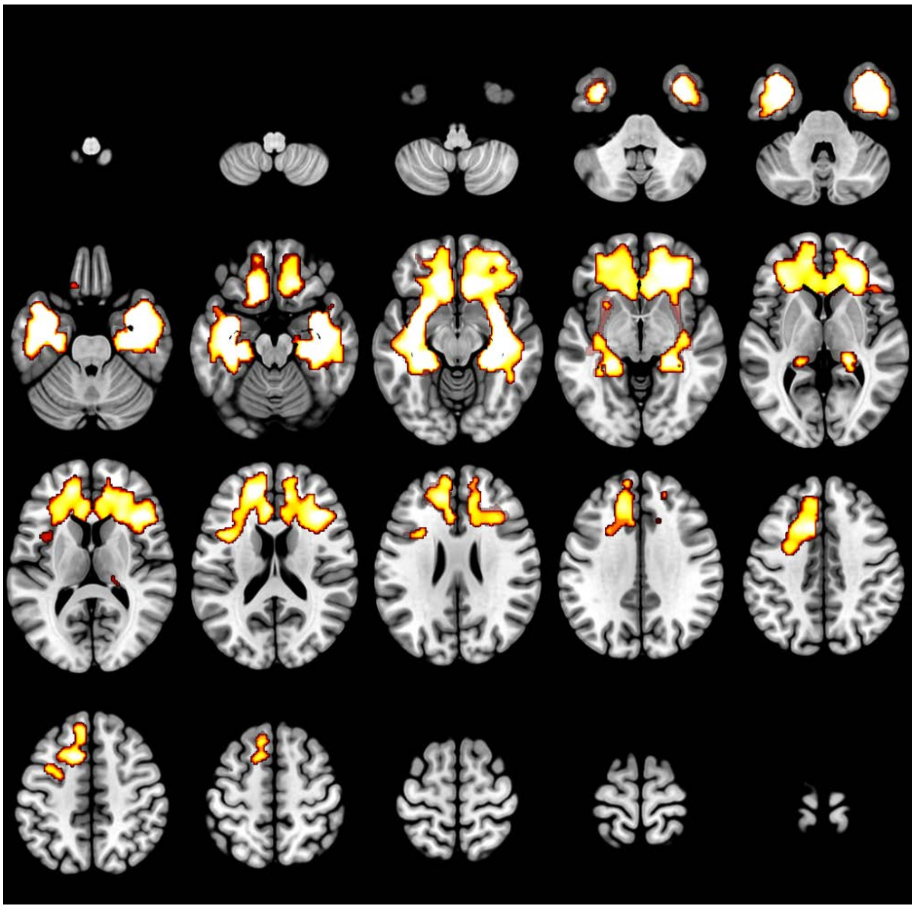

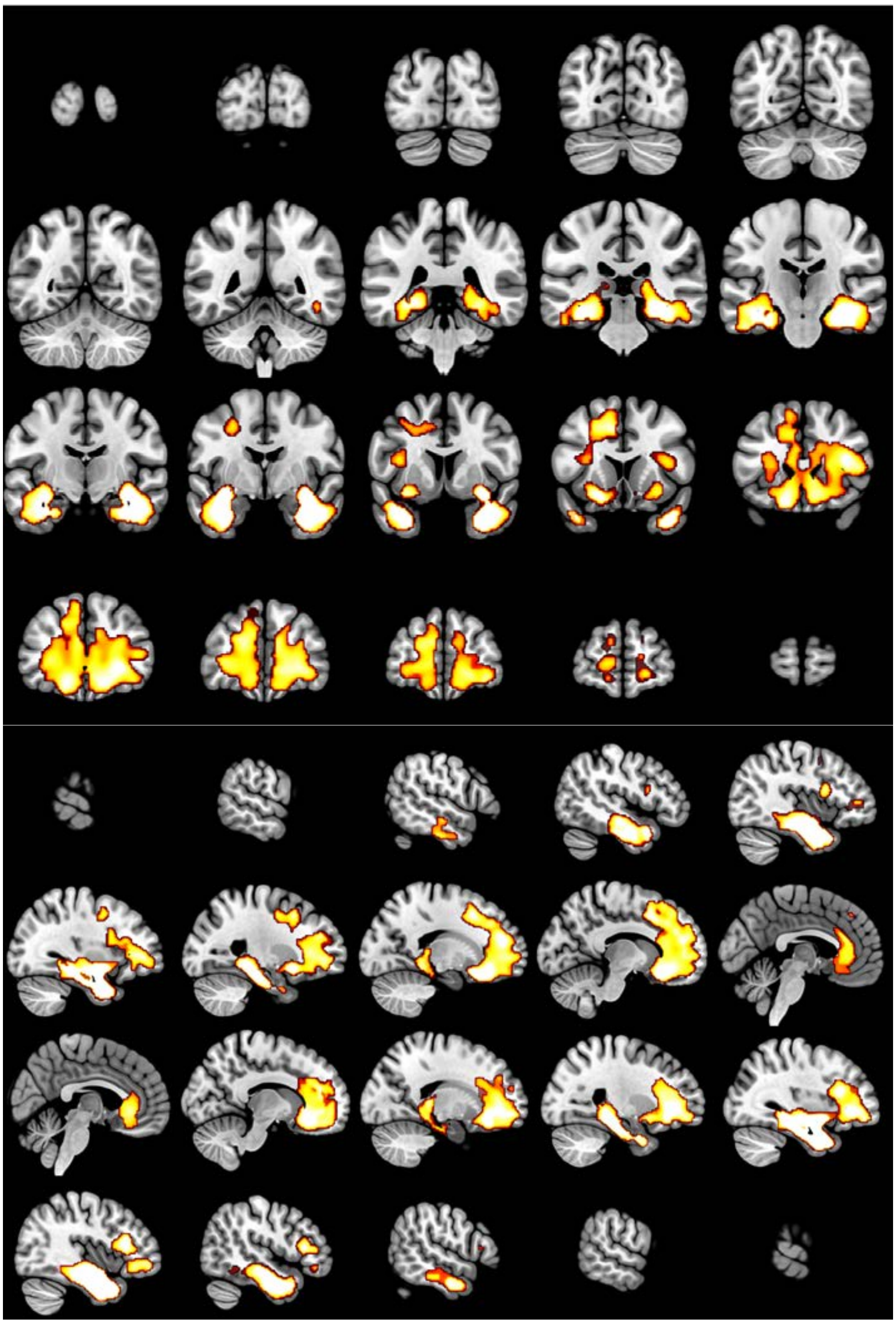

Figure 7: Grey Matter Correlates of Carer Rated Change in Behaviour (Component 3-ve)

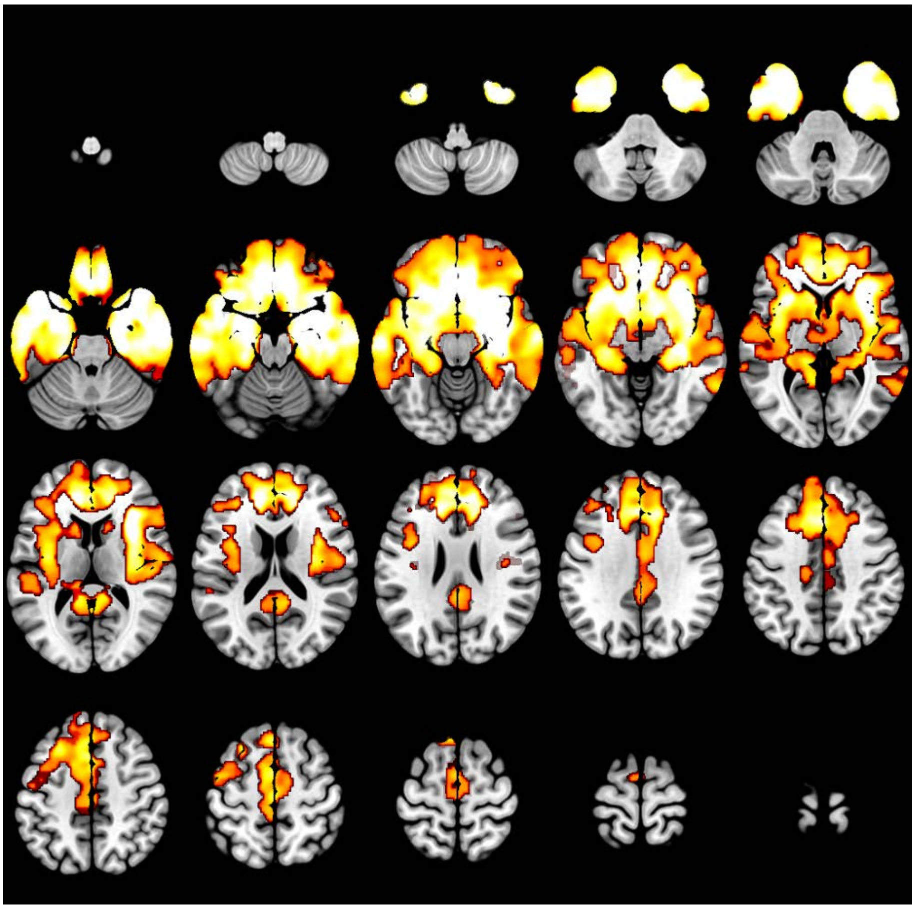

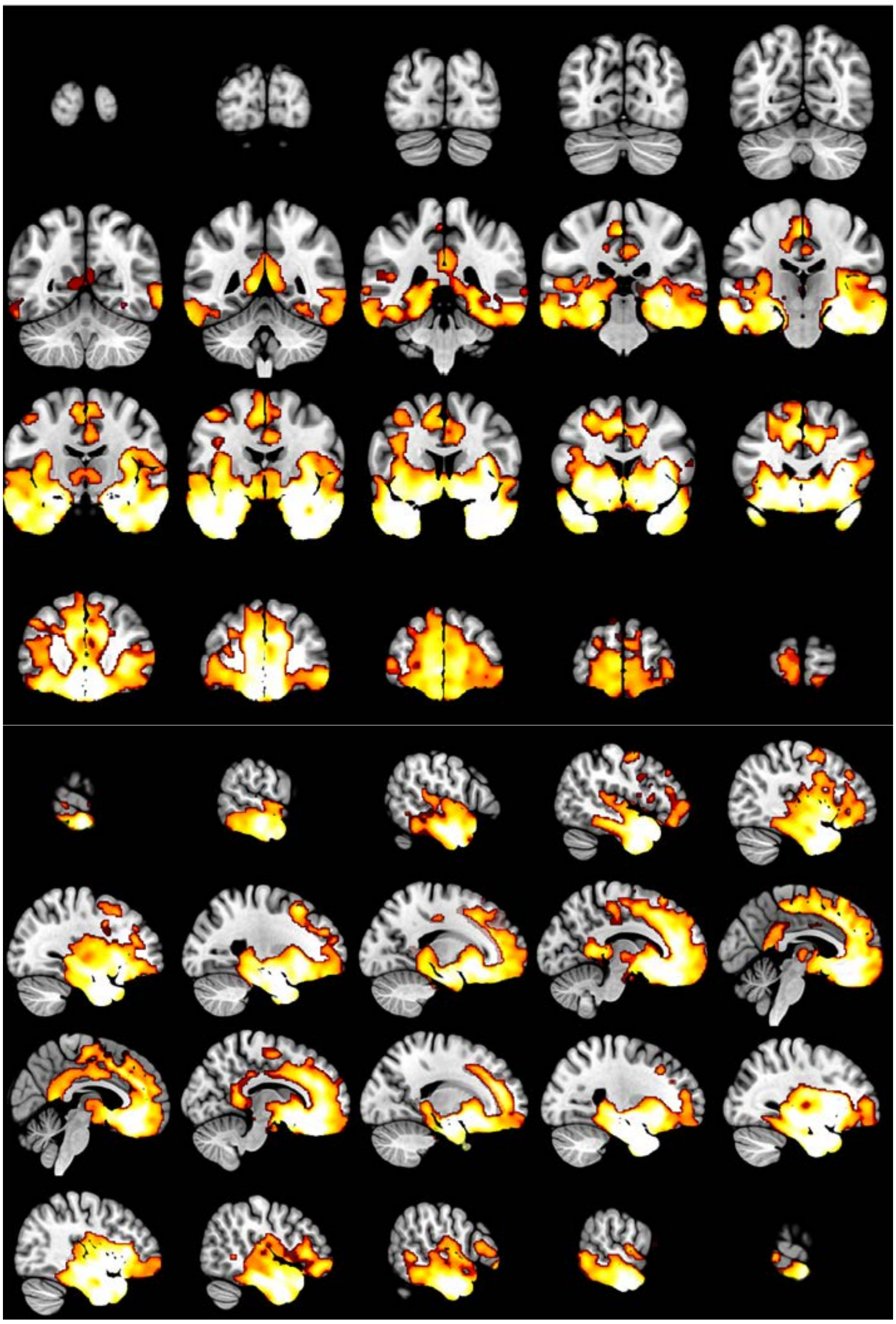

Figure 8: Grey Matter Correlates of Impulsive Behaviours (Component 4-ve)

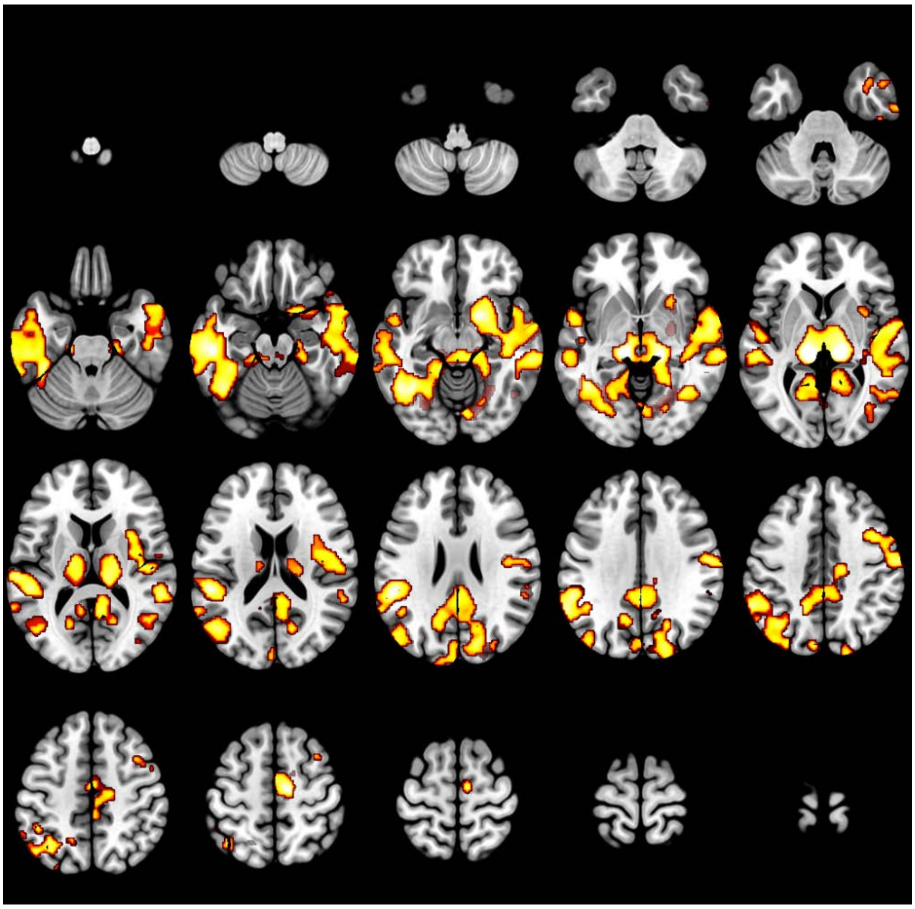

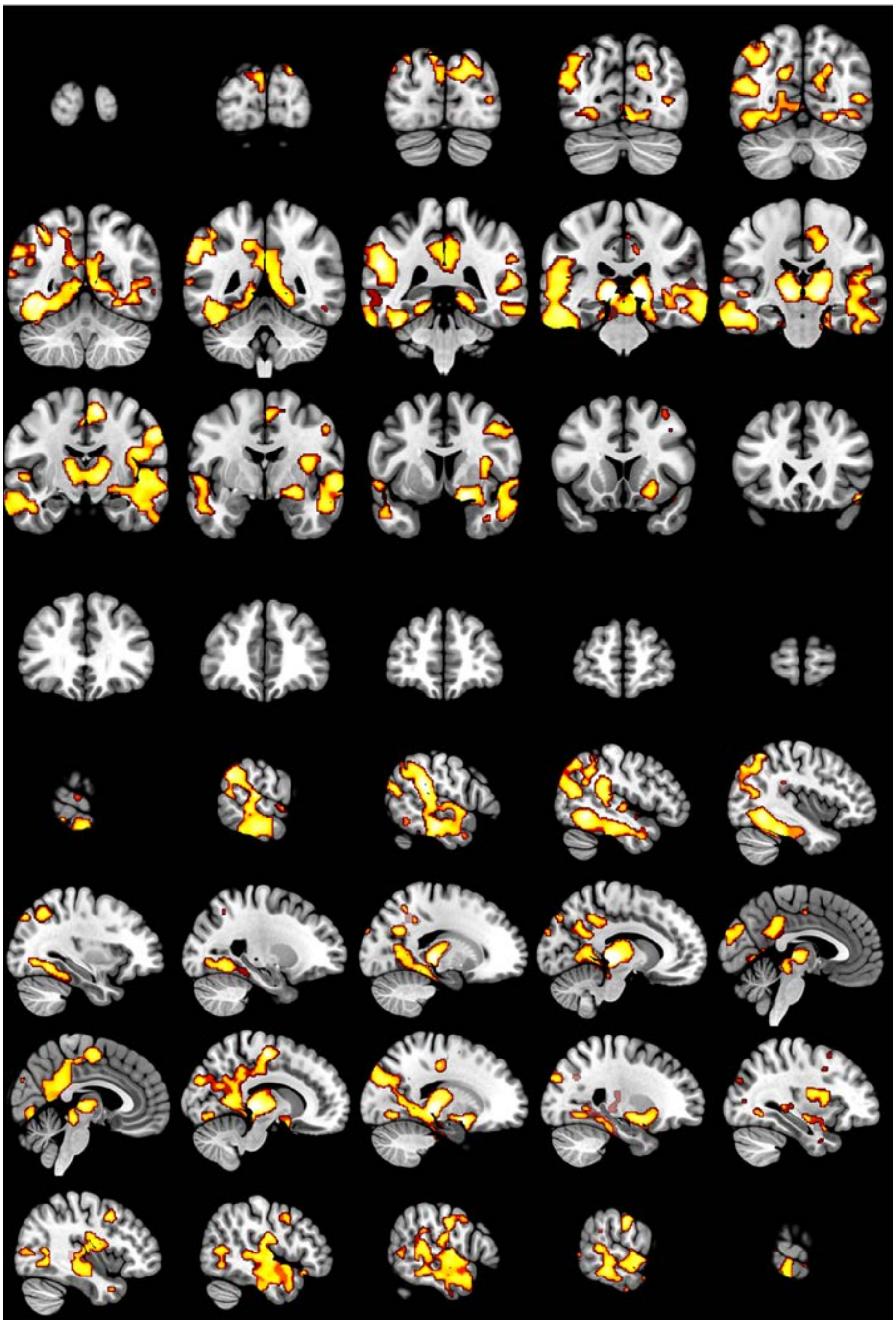

Figure 9: White Matter Correlates of Stop Signal Performance (Component 7+ve)

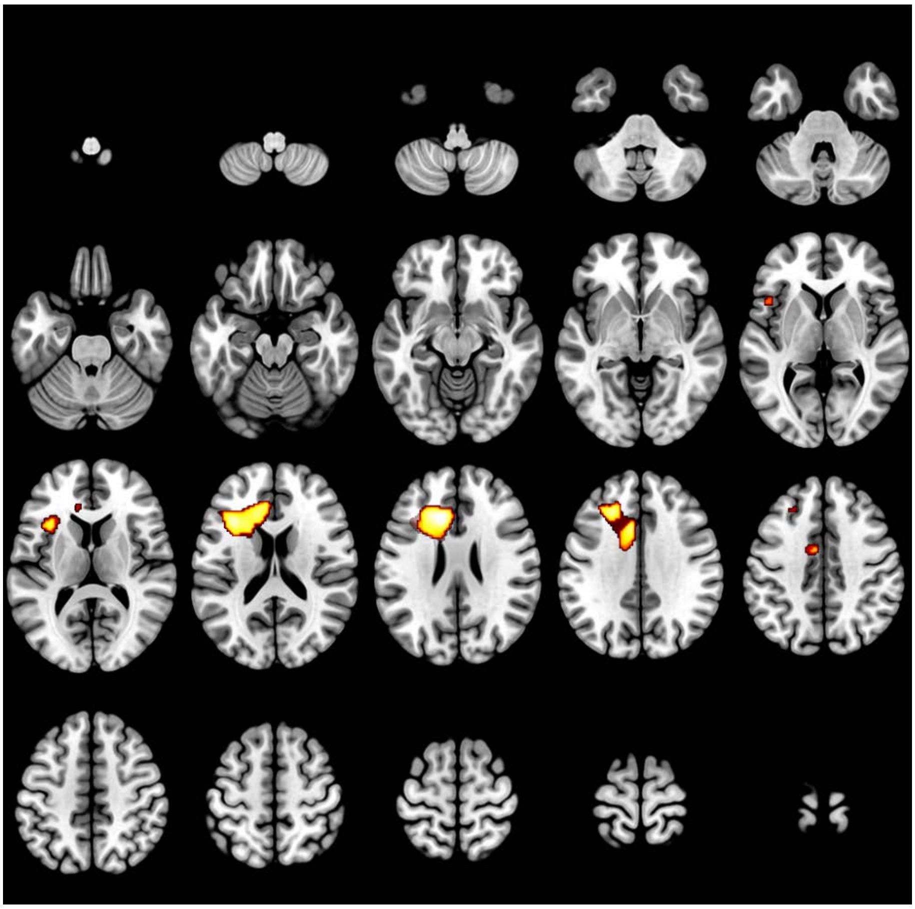

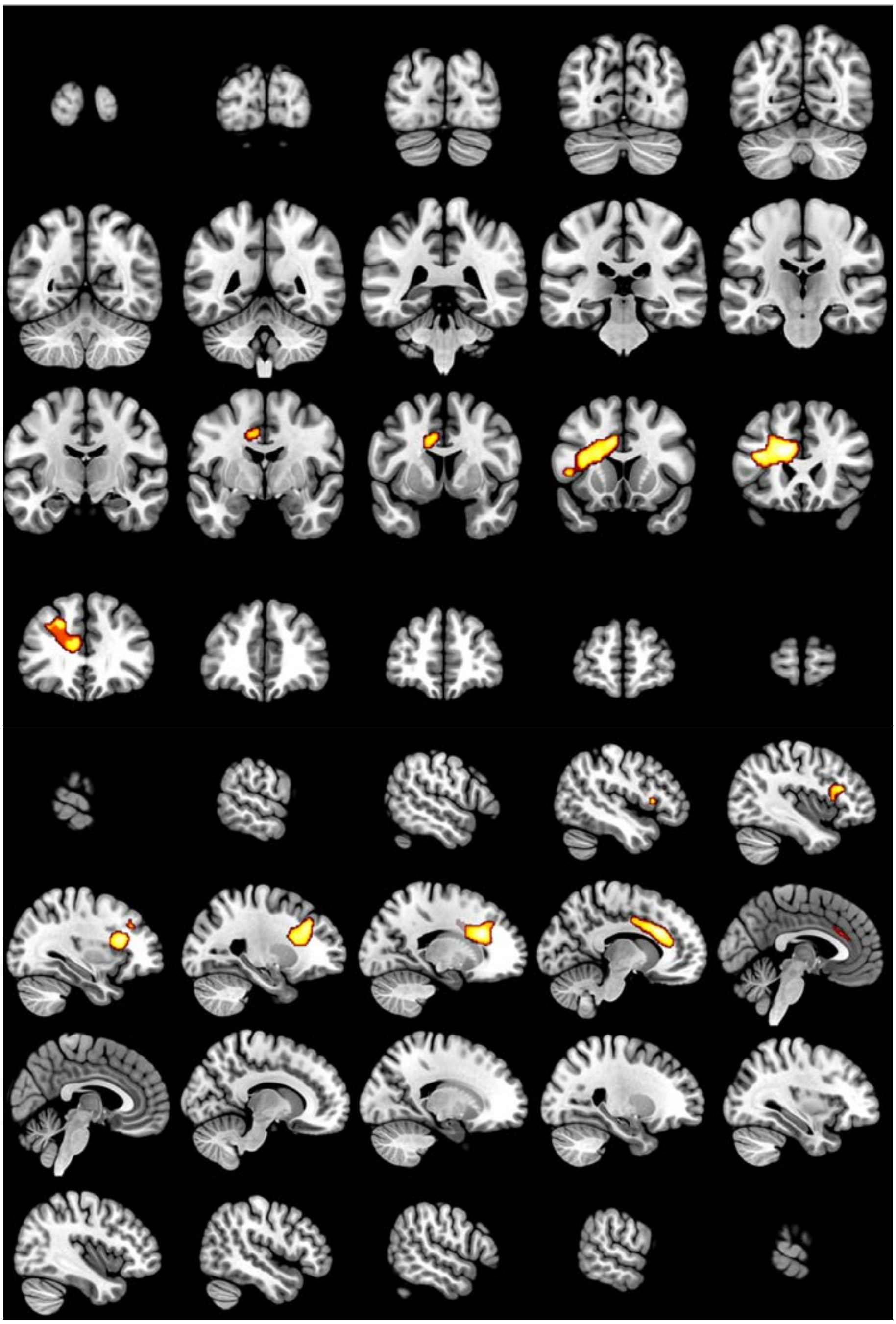

Figure 10: Grey Matter Correlates of Stop Signal Performance (Component 7+ve)

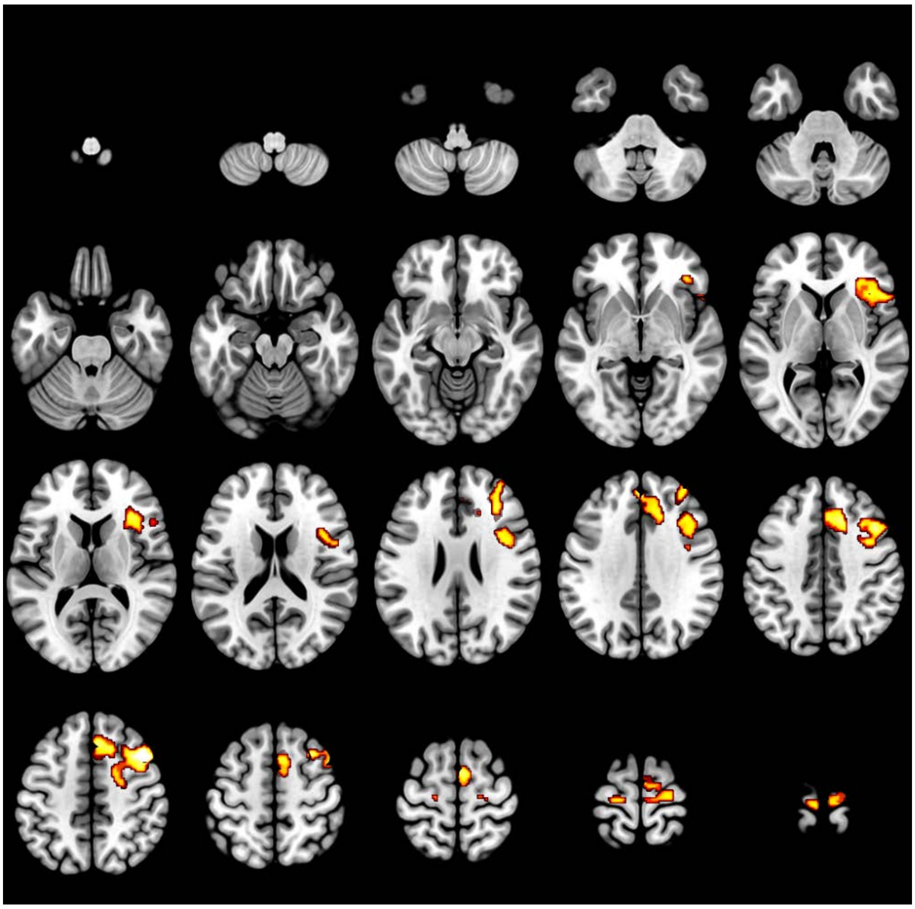

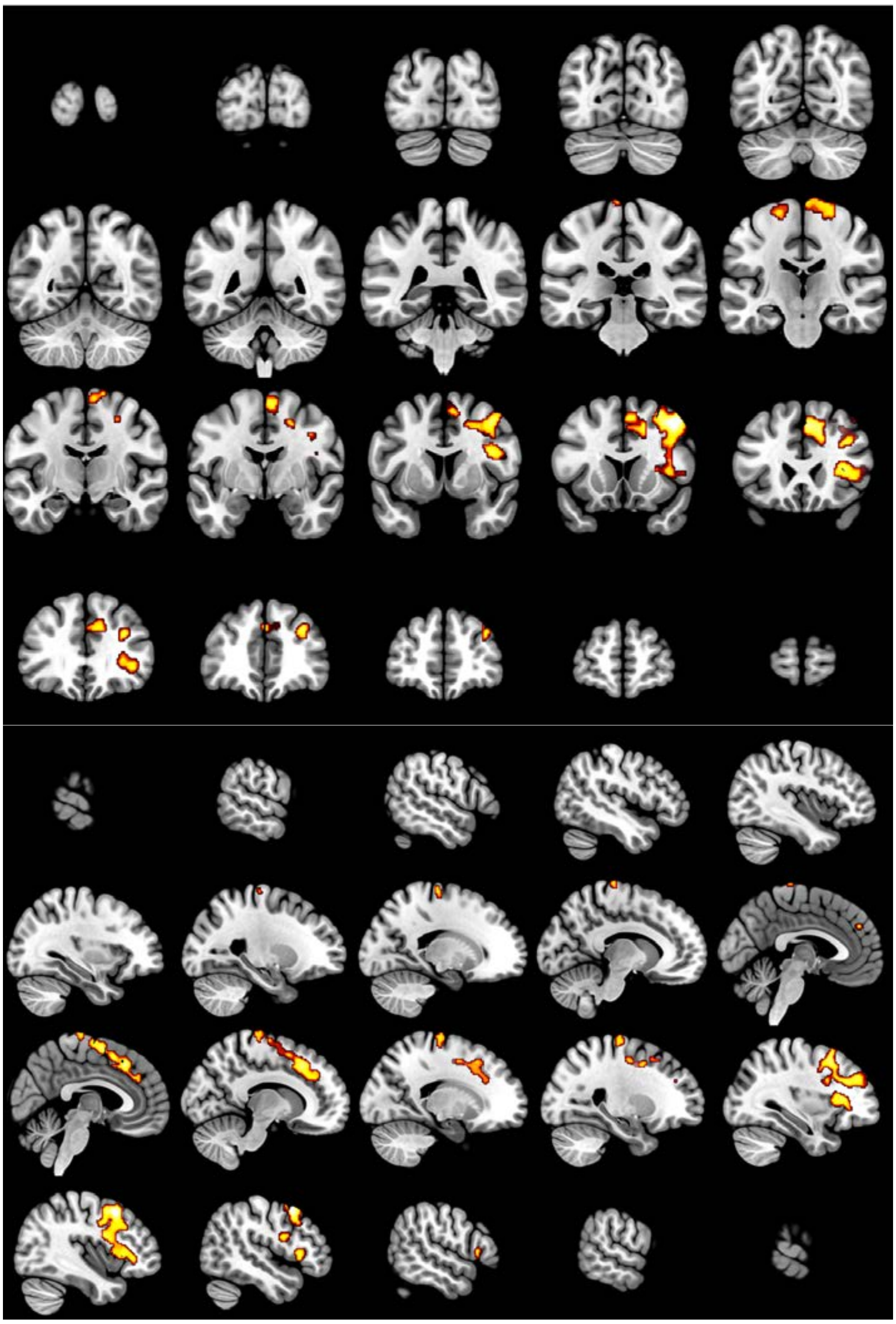

Supplement: Supplementary Data [file awx101_Supp.pdf]
